# Development and validation of a risk prediction model for diabetic kidney disease in patients with diabetic retinopathy

**DOI:** 10.3389/fendo.2025.1499866

**Published:** 2025-05-05

**Authors:** Mengsha Yin, Wenke Dong, Linan Ren, Mingyue Han, Guixia Wang, Yao Wang, Xiaokun Gang

**Affiliations:** ^1^ Department of Endocrinology and Metabolism, The First Affiliated Hospital of Jilin University, Changchun, China; ^2^ Department of Medical Imaging Technology, Changzhi Medical College, Changzhi, China; ^3^ Department of Endocrinology and Metabolism, The Second Affiliated Hospital of Jilin University, Changchun, China; ^4^ Department of Orthopedics, The Second Affiliated Hospital of Jilin University, Changchun, China

**Keywords:** diabetic kidney disease, diabetic retinopathy, type 2 diabetes mellitus, risk factors, nomogram

## Abstract

Diabetic retinopathy (DR) and diabetic kidney disease (DKD) are the most common microvascular complications associated with type 2 diabetes mellitus (T2DM). However, the occurrence of DR and DKD is not parallel. The aim of our study is to identify the risk factors for combining DKD in T2DM patients with pre-existing DR and construct a nomogram predictive model to identify high-risk patients with DR combined with DKD. We retrospectively reviewed 683 T2DM patients with DR from March 2017 to March 2023. The patients were divided into the DR group and the DR combined with DKD group. The hold-out method was used to randomly divide all subjects into a training set (70%) and a validation set (30%). Using multivariate logistic regression, we identified eight independent risk factors: fibrinogen (FIB), albumin (ALB), atherogenic index of plasma (AIP), low-density lipoprotein cholesterol (LDL-C), body mass index (BMI), classification of DR, gender, and history of hypertension. These factors were used to construct the nomogram prediction model. The model’s discriminative ability was assessed using receiver operating characteristic (ROC) curve analysis, yielding an area under the curve (AUC) of 0.780 (95% CI: 0.736-0.823) in the training set and 0.739 (95% CI: 0.668-0.809) in the validation set. Calibration curves and decision curve analysis (DCA) further demonstrated the model’s clinical utility. Additionally, to explore potential genetic predisposition, single nucleotide polymorphism (SNP) genotyping analysis was conducted on a subset of 50 randomly selected patients (25 from each group). The results suggested that the rs6591190 and rs12146493 loci of the AP5B1 gene might be associated with an increased susceptibility to DKD in patients with DR, warranting further investigation. In summary, our nomogram represents a valuable tool for identifying T2DM patients with DR who are at high risk for developing DKD.

## Introduction

Diabetes mellitus (DM) is a prevalent chronic progressive disease worldwide. Currently, approximately one in ten adults globally suffers from DM, with 90% of cases being type 2 diabetes mellitus (T2DM) (1, 2). The rising incidence of T2DM correlates with an annual increase in microvascular complications. Among these, diabetic kidney disease (DKD) and diabetic retinopathy (DR) stand out as the most common in T2DM patients (3, 4).

Epidemiological studies have highlighted a close association between DKD and DR due to their similar structural and physiological changes (5, 6). However, in the real world, DR and DKD can manifest independently, and their progression may not always align (7). For instance, some DM patients experience retinopathy without concurrent kidney impairment, suggesting varying pathogenic mechanisms and risk factors. Notably, C-peptide levels exert differing effects on DKD and DR. In a real-world observational study, C-peptide was observed to promote DKD while conferring protection against DR (8). Furthermore, genetic susceptibility likely plays a pivotal role in disease pathogenesis (9–11). Investigations employing single nucleotide polymorphisms (SNPs) as genetic markers have identified susceptibility genes (12). Recent studies have underscored distinct genetic predispositions for both DKD and DR, reflecting differences in their genetic backgrounds and susceptibility genes (11, 13–15). Despite these findings, the specific risk factors contributing to the coexistence of DKD in patients with pre-existing DR remain inadequately explored. To address this gap, our study systematically analyzed the clinical differences between T2DM patients with DR alone and those with DR combined with DKD, aiming to identify independent risk factors for DKD in this population. Based on these predictors, we developed a nomogram model to provide individualized risk assessment. Furthermore, we conducted SNP genotyping analysis to explore potential genetic predispositions associated with the combined presence of DKD and DR. To our knowledge, this is the first study to construct a predictive model specifically for DKD in T2DM patients with DR while incorporating genetic susceptibility analysis. Our findings enhance the understanding of the interplay between these diabetic microvascular complications and provide a foundation for early identification and targeted interventions in high-risk populations.

## Materials and methods

### Study design and participants

We collected data from 683 T2DM patients diagnosed with DR who were hospitalized in the Endocrinology and Metabolism Department of the First Hospital of Jilin University from March 2017 to March 2023. Patients were grouped based on 24-hour urinary microalbumin and/or estimated glomerular filtration rate (eGFR): those with 24-hour urinary microalbumin ≥ 30 mg and/or eGFR < 60 ml/min were classified into the DR combined with DKD group, while others were classified into the DR group. Diagnosis of T2DM followed criteria outlined in the “Guidelines for the Prevention and Control of Type 2 Diabetes in China (2019)” (16). The diagnostic criteria for DR were based on the International Clinical Grading Standards (2002). Exclusion criteria included (1): acute complications (diabetic ketoacidosis, hyperosmolar hyperglycemic syndrome, severe infection, lactic acidosis, etc.) (2); type 1 diabetes or secondary diabetes (3); T2DM with other retinal vascular diseases complicated by macular edema (4); other retinal diseases (e.g., age-related macular degeneration, uveitis, hereditary retinal diseases) (5); acute or chronic nephritis, nephrotic syndrome, urinary infections, renal tumors, renal vascular diseases (6); use of drugs affecting urinary protein excretion or nephrotoxic drugs.

### Data collection

General data collected included demographic characteristics, clinical information, and laboratory data such as blood routine, coagulation routine, urine routine, blood glucose-related indicators, liver function, kidney function, four lipid panel items, blood ions, thyroid function, as well as 24-hour urinary protein and urinary microalbumin levels. All these data were measured at the time of hospital admission. DR staging was performed using fundus photography and independently assessed by two ophthalmologists. Cases were classified according to the International DR Staging Standards (2003) into non-proliferative diabetic retinopathy (NPDR) and proliferative diabetic retinopathy (PDR). Hypertension was defined as a systolic blood pressure ≥ 140 mmHg and/or diastolic blood pressure ≥ 90 mmHg, measured on three separate occasions, or the current use of antihypertensive medication, or a documented history of hypertension. History of coronary heart disease was defined as a prior diagnosis of coronary heart disease. History of stroke was defined as a prior cerebrovascular event, confirmed by clinical diagnosis or supporting neuroimaging evidence. History of fatty liver was defined as a prior diagnosis of fatty liver disease, confirmed by imaging studies, abnormal biochemical markers, or clinical assessment by a physician. Smoking history was defined as current smoking (≥1 cigarette per day for at least 6 months) or former smoking (having smoked for ≥6 months but quit for at least 12 months). Alcohol consumption history was defined as current drinking (≥1 standard drink per week for the past 6 months) or former drinking (regular consumption for ≥6 months but abstinent for at least 12 months). Fasting blood samples were collected in the morning following an overnight fast of at least 8 hours. A family history of T2DM was defined as a history of T2DM in at least one parent or sibling.

### Correlation variable definitions

Body mass index (BMI) = weight (kg)/height (m)^2; Neutrophil-to-lymphocyte ratio (NLR) = neutrophil absolute value (NE)/lymphocyte absolute value (LY); Lymphocyte-to-monocyte ratio (LMR) = LY/monocyte absolute value (MO); Platelet-to-lymphocyte ratio (PLR) = platelet/LY; Residual cholesterol (mmol/L) = cholesterol (TC) – low-density lipoprotein cholesterol (LDL-C) – high-density lipoprotein cholesterol (HDL-C); Atherogenic index (AI) = (TC - HDL-C)/HDL-C; Atherogenic plasma index (API) = LDL-C/HDL-C; Atherogenic index of plasma (AIP) = log(triglyceride/HDL-C) (the unit of all lipid indicators is mmol/L); TyG index = ln[triglyceride (mg/dl) * plasma glucose (mg/dl)/2].

### SNPs data collection

To explore potential genetic susceptibility to DR combined with DKD, twenty-five baseline-matched whole blood samples were selected from each group for SNPs genotyping.

- The main instruments, equipment, experimental reagents, and consumables are listed in **Supplementary Tables 1** and **2**.
- The candidate genes for this study were determined through a comprehensive review of the literature (**Table 1**).
- The selection of SNPs loci in candidate genes was based on two primary sources: functional SNPs loci identified from the NCBI dbSNP database, focusing on gene functional regions such as Exon, Promoter, 5’ UTR, and 3’ UTR, and literature-derived SNPs identified through Google Scholar and databases such as GWAS Catalog, GWAS Central, and GWAS Atlas. SNPs with a minor allele frequency (MAF) < 0.01 in the Chinese Han population in Beijing based on data from the 1000 Genomes database were excluded. Predicted functionality of selected SNPs was assessed using http://snpinfo.niehs.nih.gov/, and linkage disequilibrium (LD) analysis was performed using http://asia.ensembl.org/Homo_sapiens/Tools/LD?db=core. The final SNPs loci of candidate genes are presented in **Table 2**.
- Genotyping: We conducted the study using whole blood samples stored at -80°C in an ultra-low temperature freezer. DNA was extracted from the blood samples using a commercial DNA extraction kit, ensuring extracted DNA met quality standards. SNPs genotyping of candidate genes was performed using Agena MassArray technology provided by Biomiao Biological Technology (Beijing) Co., Ltd. Initially, polymerase chain reaction (PCR) amplified DNA sequences, followed by a reaction system incorporating four dideoxyribonucleotide triphosphates and single-base extension primers to amplify PCR products. SNPs genotyping was conducted using a time-of-flight mass spectrometry system, distinguishing SNPs alleles based on molecular weight differences.

**Table 1:** The candidate genes.

Gene name	Genetic locus information	Coding protein
AP5B1	Chr11: 65773898.65780976	AP-5 complex subunit beta-1
TENM2	Chr5: 166979029.168264157	Teneurin-2
CUBN	Chr10: 16823966.17129811	Cubilin
UMOD	Chr16: 20333051.20356301	Uromodulin
PTPRO	Chr12: 15322508.15598331	Receptor-type tyrosine- protein phosphatase O

**Table 2:** The SNPs loci of candidate genes.

Gene	SNPs locus	Location	MAF	Potential function prediction
AP5B1	rs4014195	‐‐	0.210	‐‐
	rs6591190	Promoter	0.422	TFBS
	rs522800	3’UTR	0.359	miRNA binding site
	rs12146493	Exon-missense	0.340	nsSNP
TENM2	rs72831309	Intron	0	‐‐
	rs1862416	Intron	0.102	‐‐
	rs3733989	3’UTR	0.243	miRNA binding site
	rs4242220	Intron	0.282	‐‐
	rs11272049	Promoter	0.490	‐‐
CUBN	rs11254238	Intron	0.150	‐‐
	rs1801239	Exon-missense	0.167	nsSNP
	rs74375025	Intron	0.130	‐‐
	rs7918972	Intron	0.374	‐‐
	rs45551835	Exon-missense	0.169	Splicing (ESE or ESS);nsSNP
	rs141640975	‐‐	0	‐‐
	rs45619139	Intron	0.206	‐‐
	rs2271462	Exon-missense	0.209	Splicing (ESE or ESS);nsSNP
	rs539606836	Intron	0	‐‐
	rs572663329	Intron	0.017	‐‐
UMOD	rs13329952	Promoter	0.102	TFBS
	rs11864909	Promoter	0.194	‐‐
	rs77924615	Intron	0.204	‐‐
	rs12922822	Promoter	0.010	TFBS
	rs34882080	Intron	0.010	‐‐
PTPRO	rs7976329	Intron	0.257	‐‐
	rs3748299	Exon-synonymous	0.340	Splicing (ESE or ESS)
	rs1050646	Exon-synonymous	0.117	Splicing (ESE or ESS)
	rs2300290	Intron	0.180	‐‐
	rs7956634	Intron	0.277	‐‐
	rs6488782	Exon-synonymous	0.136	Splicing (ESE or ESS)

MAF, minor allele frequency; TFBS, transcription factor binding site; miRNA, microRNA.

### Statistical analysis

The measurement data were described as means ± standard deviations if they followed a normal distribution and as medians and interquartile range (P25, P75) otherwise. For categorical data, frequencies and percentages (%) were used. To compare measurement data between two groups, a t-test was used if the data followed a normal distribution and had equal variances; otherwise, the Wilcoxon rank-sum test was used. Categorical data were compared using the χ^2^ test or fisher’s exact test. Univariate logistic regression analysis was used to analyze the variables. Variables with statistically significant results, combined with professional knowledge, were included in the multivariate logistic regression model. The final factors were screened by forward stepwise regression method, and the prediction model was constructed and visualized as a nomogram using the rms package in R. Model discrimination was evaluated by calculating the area under the receiver operating characteristic (ROC) curve (AUC), with ROC curves generated using the pROC package. Model calibration was evaluated using the Hosmer-Lemeshow (H-L) test and bootstrap-corrected calibration curves. Calibration curves were generated using the rms::calibrate function with 1,000 bootstrap resamples to correct overfitting. Deviations from ideal calibration were quantified by mean absolute error (MAE). Decision curve analysis (DCA) implemented through the rmda package and was performed to quantify the net clinical benefit of the model across a range of threshold probabilities, with comparisons made to “treat all” and “treat none” strategies. To ensure the model’s generalizability, the dataset was randomly split into a training set (70%) and a validation set (30%). Model validation was performed using the validation dataset, with AUC, calibration curves, and DCA applied independently. The data processing software used included IBM SPSS 25.0 and R software (version 4.2.0), with a significance level of α = 0.05.

The SNPStats (https://www.snpstats.net) was used to analyze SNPs-related data. DR with or without DKD was the dependent variable, while SNPs locus alleles and genotypes were the independent variables in a binary logistic regression analysis, with *P* < 0.05 considered statistically significant. Genotype analysis was based on five genetic models: co-dominant, dominant, recessive, over-dominant, and log-additive models. Specifically, if AA represents wild-type homozygous, AC represents heterozygous variant, and CC represents homozygous variant, the models are as follows: Co-dominant model: CC vs AA; AC vs AA; Dominant model: (AC+CC) vs AA; Recessive model: CC vs (AC+AA); Over-dominant model: (AA+CC) vs AC; Log-additive model: CC vs AA.

## Results

### Baseline characteristics of participants

Using the hold-out method, data from 683 patients were randomly divided into a training set and a validation set in a 7:3 ratio, resulting in 478 patients in the training set and 205 patients in the validation set. Comparative analysis of the training and validation sets showed that both general and laboratory data were comparable (*P* > 0.05), as shown in **Supplementary Table 3**.

### Univariate and multivariate analyses of risk predictors

Univariate logistic regression results indicated that gender, classification of DR, history of hypertension, history of stroke, smoking history, duration of smoking, systolic blood pressure, BMI, fibrinogen (FIB), albumin (ALB), TC, HDL-C, LDL-C, residual cholesterol, AI, API, AIP, TyG index, and free triiodothyronine were statistically significant (*P* < 0.05), as shown in **Supplementary Table 4**. These factors were then included in a multivariate logistic regression analysis to identify independent risk factors for DR combined with DKD. The results showed that FIB (OR = 1.52, 95% CI: 1.11-2.08), ALB (OR = 0.92, 95% CI: 0.87-0.97), AIP (OR = 3.96, 95% CI: 1.91-8.24), LDL-C (OR = 1.43, 95% CI: 1.11-1.84), BMI (OR = 1.08, 95% CI: 1.01-1.16), classification of DR (OR = 2.81, 95% CI: 1.32-5.96), gender (OR = 0.36, 95% CI: 0.22-0.60), and history of hypertension (OR = 2.73, 95% CI: 1.73-4.30) were independent risk factors for the DR combined with DKD group (*P* < 0.05), as shown in **Table 3**.

**Table 3:** Multivariate logistic regression analysis of training set.

Characteristic	Beta	S.E.	Z	OR (95%CI)	*P* value
FIB (g/L)	0.42	0.16	2.64	1.52(1.11-2.08)	0.008*
ALB (g/L)	-0.09	0.03	-3.07	0.92(0.87-0.97)	0.002*
AIP	1.38	0.37	3.69	3.96(1.91-8.24)	<0.001*
LDL-C (mmol/L)	0.36	0.13	2.78	1.43(1.11-1.84)	0.005*
BMI (kg/m^2^)	0.08	0.04	2.12	1.08(1.01-1.16)	0.034*
Classification of DR (PDR vs. NPDR)	1.03	0.38	2.69	2.81(1.32-5.96)	0.007*
Gender (female vs. male)	-1.01	0.25	-3.97	0.36(0.22-0.60)	<0.001*
History of hypertension (yes vs. no)	1.00	0.23	4.32	2.73(1.73-4.30)	<0.001*

S.E., Standard error; FIB, fibrinogen; ALB, albumin; AIP, atherogenic index of plasma;LDL-C, low density lipoprotein cholesterol; BMI, body mass index; DR, diabetic retinopathy; PDR, proliferative diabetic retinopathy; NPDR, non-proliferative diabetic retinopathy. *P < 0.05, with statistical difference.

### Construction of the nomogram prediction model

**Figure 1:**
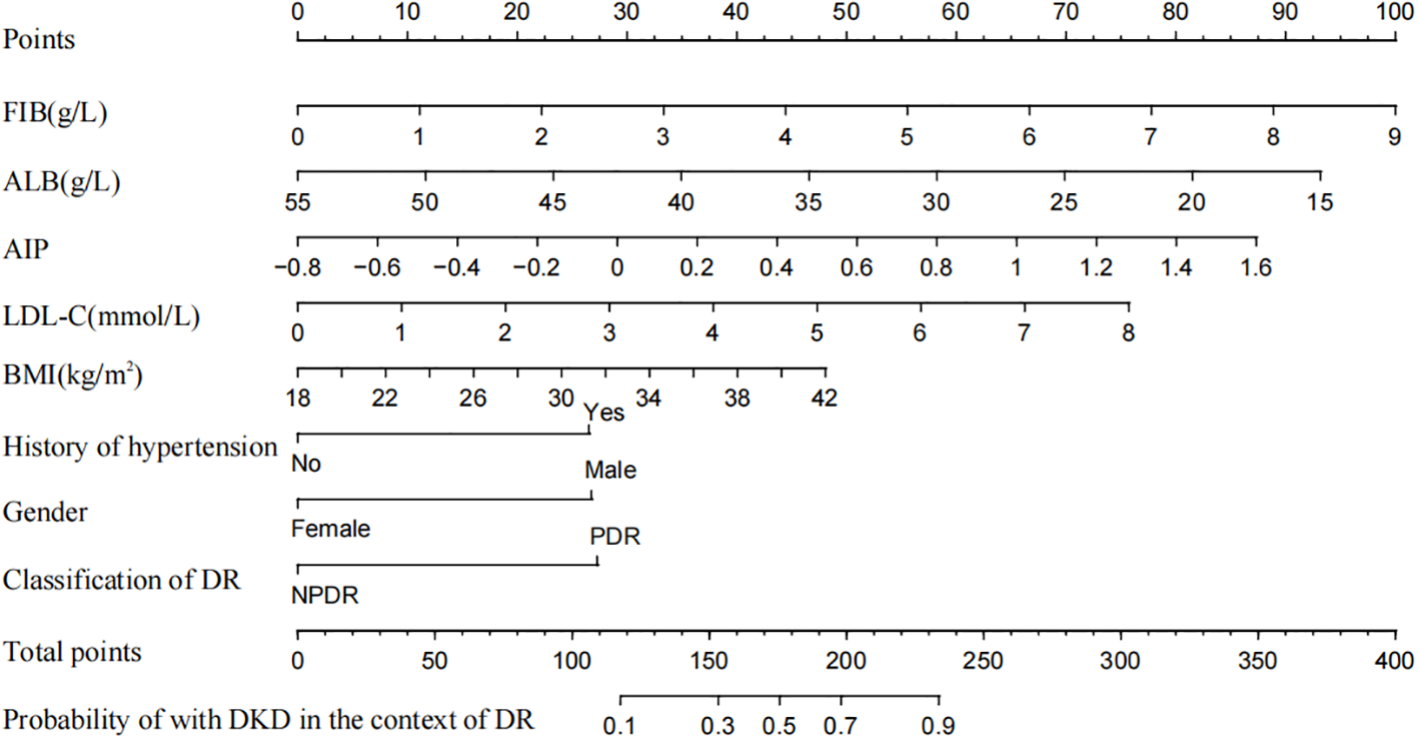
Nomogram to predict the risk of DKD for patients with DR. FIB, fibrinogen; ALB, albumin; AIP, atherogenic index of plasma; LDL-C, low density lipoprotein cholesterol; BMI, body mass index; DR, diabetic retinopathy; PDR, proliferative diabetic retinopathy; NPDR, non-proliferative diabetic retinopathy.

The indicators screened in the multivariate regression were used to construct the prediction model, visualized as a nomogram, as shown in **Figure 1**. By summing the scores corresponding to each predictive indicator, the total score was obtained. The risk value corresponding to the total score indicates the probability of a patient with DKD in the context of DR.

### Evaluation and validation of the prediction model

**Figure 2:**
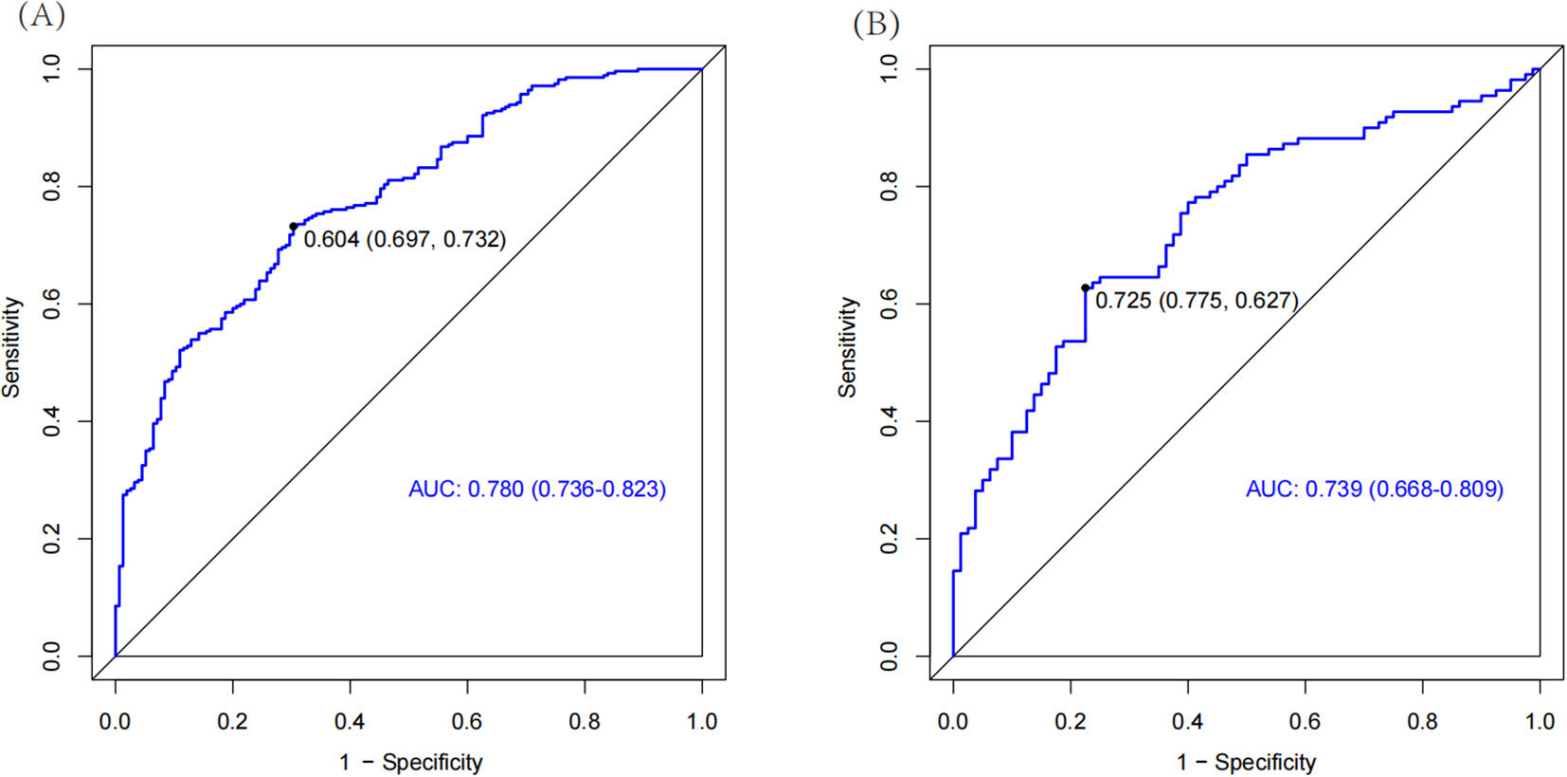
The ROC curves for training set **(A)** and validation set **(B)**. The part below the blue line is the AUC of the model. AUC, area under curve.

**Figure 3:**
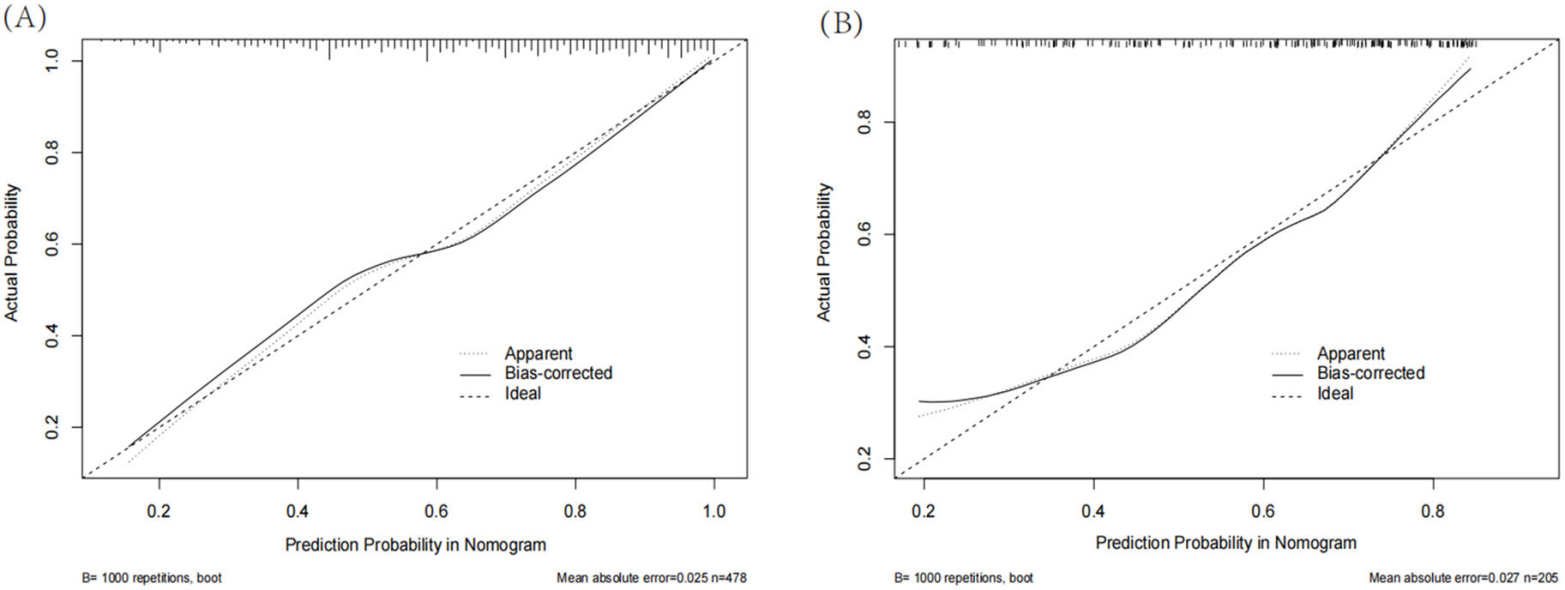
Calibration curves for training set **(A)** and validation set **(B)**. The solid line represents the model after calibration. The closer the calibration curve of the model is to the ideal line, the better the model’s prediction accuracy.

**Figure 4:**
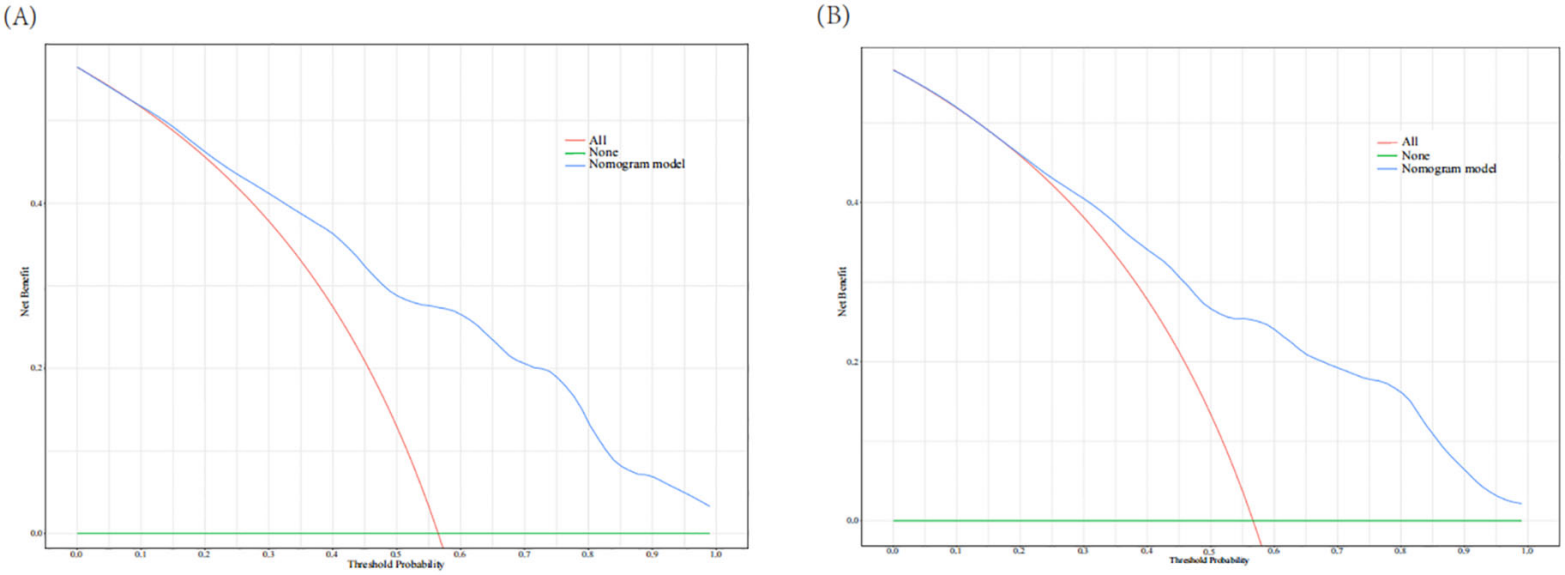
Decision curve analysis for training set **(A)** and validation set **(B)**. The red line indicates that all patients experienced DKD and the green line represents that no patients experience DKD. The blue line represents the nomogram model. The curves show that the model is clinically beneficial across a relatively wide range of threshold probabilities.

The AUC of the nomogram model in the training set was 0.780 (95% CI: 0.736-0.823). When distinguishing between high and low risk using the optimal predictive risk cutoff value of 0.604, the specificity was 0.697 and the sensitivity was 0.732, indicating good discrimination (**Figure 2A**). In the validation set, the AUC was 0.739 (95% CI: 0.668-0.809). Using 0.725 as the cutoff for high and low risk stratification, the specificity was 0.775 and the sensitivity was 0.627 (**Figure 2B**). In both the training and validation sets, calibration curves showed good consistency between the predicted and ideal curves. After 1,000 bootstrap resampling iterations, the mean absolute errors were 2.5% and 2.7%, respectively (**Figures 3A, B**). The DCA curves for both the training and validation sets demonstrated that this model provides good net benefits for clinically predicting the risk of DKD in patients with DR (**Figures 4A, B**).

### SNPs-related research results

The results of SNPs genotyping of the submitted blood samples are shown in **Supplementary Table 5**. Hardy-Weinberg (H-W) equilibrium analysis of the different sample groups showed that all 27 SNPs loci conformed to the H-W equilibrium law (i.e., *HWP* value ≥ 0.001).

Whether the presence of DKD in patients with DR as the dependent variable and the alleles (variant genes) of each SNPs locus as the independent variables, binary logistic regression was performed to identify susceptible alleles. The results revealed that the alleles of AP5B1’s rs6591190 and rs12146493 were statistically significant (*P* < 0.05) (**Table 4**).

**Table 4:** The alleles logistic regression analysis of DR combined with DKD.

Gene	SNPs locus	Minor allele	OR(95%CI)	*P* value
AP5B1	rs4014195	G	0.50 (0.17-1.47)	0.206
	rs6591190	G	3.36 (1.21-9.32)	0.019*
	rs522800	C	0.73 (0-inf)	0.999
	rs12146493	A	0.41 (0.17-0.99)	0.049*
TENM2	rs3733989	G	1.36 (0.41-4.50)	0.617
	rs1862416	C	0.18 (0.01-1.58)	0.121
	rs4242220	G	1.24 (0.49-3.08)	0.643
	rs11272049	G	0.84 (0.38-1.83)	0.664
CUBN	rs11254238	C	0.99 (0.35-2.78)	0.982
	rs74375025	A	0.73 (0-inf)	0.999
	rs7918972	G	0.95 (0.41-2.19)	0.910
	rs1801239	C	0.73 (0-inf)	0.999
	rs45619139	G	0.73 (0-inf)	0.999
	rs45551835	A	1.82 (0.26-12.54)	0.546
	rs572663329	G	0.73 (0-inf)	0.999
	rs2271462	T	1.78 (0.58-5.47)	0.312
UMOD	rs13329952	C	0.80 (0.23-2.75)	0.717
	rs11864909	T	0.39 (0.12-1.31)	0.128
	rs77924615	A	0.44 (0.14-1.38)	0.159
	rs34882080	G	0.73 (0-inf)	0.999
	rs12922822	T	0.73 (0-inf)	0.999
PTPRO	rs7976329	C	1.37 (0.56-3.36)	0.489
	rs2300290	A	1.18 (0.44-3.15)	0.737
	rs3748299	A	1.01 (0.46-2.23)	0.987
	rs1050646	C	0.94 (0.31-2.85)	0.918
	rs7956634	C	0.73 (0-inf)	0.999
	rs6488782	A	0.73 (0.25-2.15)	0.570

*P < 0.05, with statistical difference.

Whether DR combined with DKD as the dependent variable and the genotypes of various SNPs loci as independent variables, binary logistic regression was performed to identify susceptible genotypes. The results showed that for the rs6591190 locus of the AP5B1 gene, in the co-dominant genetic model, the heterozygous GC genotype had a higher risk of DKD compared to the homozygous CC genotype (OR = 3.45, 95% CI: 1.07-10.43, *P* = 0.008), and the variant GG genotype had a higher risk of DKD compared to the homozygous CC genotype (OR = 3.67, 95% CI: 1.05-11.87, *P* = 0.008). In the dominant genetic model, the GC and GG genotypes had a higher risk of DKD compared to the homozygous CC genotype (OR = 3.29, 95% CI: 1.80-8.12, *P* = 0.002). In the over-dominant genetic model, compared to the CC and GC genotypes, the heterozygous GC genotype had a higher risk of DKD (OR = 3.88, 95% CI: 1.15-13.04, *P* = 0.023). In the additive model, the variant GG genotype had a higher risk of DKD compared to the homozygous CC genotype (OR = 3.36, 95% CI: 1.21-9.32, *P* = 0.011). For the rs12146493 locus of the AP5B1 gene, in the additive genetic model, the variant AA genotype had a lower risk of DKD compared to the homozygous GG genotype (OR = 0.41, 95% CI: 0.16-0.98, *P* = 0.038), as shown in **Table 5**. The remaining SNPs loci were not statistically significant (*P* > 0.05) (**Supplementary Table 6**).

**Table 5:** The Genotype logistic regression analysis of DR combined with DKD.

Gene	SNPs locus	Genetic model	Genotype	OR (95%CI)	*P* value
AP5B1	rs6591190	Co-dominant	C/C	1	0.008*
			G/C	3.45 (1.07-10.43)	
			G/G	3.67 (1.05-11.87)	
		Dominant	C/C	1	0.002*
			G/C-G/G	3.29 (1.80-8.12)	
		Recessive	C/C-G/C	1	0.480
			G/G	1.78 (0.35-8.96)	
		Over-dominant	C/C-G/G	1	0.023*
			G/C	3.88 (1.15-13.04)	
		Log-additive	C/C	1	0.011*
			G/G	3.36 (1.21-9.32)	
AP5B1	rs12146493	Co-dominant	G/G	1	0.110
			A/G	0.49 (0.14-1.72)	
			A/A	0.13 (0.01-1.24)	
		Dominant	G/G	1	0.086
			A/G-A/A	0.36 (0.11-1.17)	
		Recessive	G/G-A/G	1	0.072
			A/A	0.18 (0.02-1.59)	
		Over-dominant	G/G-A/A	1	0.600
			A/G	0.73 (0.22-2.39)	
		Log-additive	G/G	1	0.038*
			A/A	0.41 (0.16-0.98)	

*P < 0.05, with statistical difference.

## Discussion

It is well known that the occurrence of diabetic microvascular complications is influenced by multiple factors. Although each risk factor may impact disease onset, no single factor is decisive. By integrating these risk factors and constructing an optimal nomogram prediction model, we can provide valuable guidance for clinical practice. To our knowledge, there is currently no risk prediction model for predicting the combination of an additional microvascular complication based on the presence of one diabetic microvascular complication. Our study is the first to construct a risk model for the combination of DKD in T2DM patients with DR.

Considering that this is a retrospective study, we excluded factors that have been confirmed to be closely related to kidney function, such as urinary protein, creatinine (Cr), blood urea nitrogen, retinol-binding protein (RBP), cystatin C, and eGFR, to ensure the causal relationship of the results. Finally, we used FIB, ALB, AIP, LDL-C, BMI, classification of DR, gender, and history of hypertension as predictors to construct the nomogram model. The AUC was 0.780 (95% CI: 0.736-0.823) in the training set and 0.739 (95% CI: 0.668-0.809) in the validation set. In addition, both the calibration curve and the DCA indicated that this model has good predictive performance and is of great significance for identifying high-risk populations. Importantly, our model is consistent with existing evidence indicating that individuals with DR are at an elevated risk of developing DKD. Furthermore, the model captures the well-established risk gradient between DR severity and DKD (16, 17), demonstrating that patients with PDR exhibit a significantly higher predicted probability of DKD compared to those with NPDR (OR=2.81). The inclusion of hypertension as a predictor reinforces the strong interplay between hypertensive status and DKD risk in DR patients, further supporting previous epidemiological findings. These results highlight the clinical utility of our nomogram in refining risk prediction for DKD in T2DM patients with DR.

In recent years, although no studies have developed prediction models specifically for DR patients with concurrent DKD, many studies have aimed to develop prediction models for the early risk of DKD in T2DM patients. Dunkler et al. (18) developed a prediction model that included five predictive factors: urine albumin creatine ratio (UACR), eGFR, albuminuria stage, age, and gender, but their study population mainly comprised patients aged 55 and older. In contrast, our study includes a broader age range, making it more applicable and incorporating relevant indicators such as weight, blood pressure, and blood lipids, comprehensively reflecting the status of metabolic syndrome. Jardine et al. (19) constructed a prediction model including seven variables (eGFR, UACR, systolic blood pressure, HbA1c, DR, gender, and education level). However, their model included T2DM patients with vascular diseases, whereas our study focuses specifically on T2DM patients with DR, providing a more detailed classification of vascular disease conditions and a more accurate model for preventing DKD in DR patients. Additionally, Xu et al. (20) constructed a prediction model that included seven risk factors: α-1-microglobulin/Cr, UACR, transferrin/Cr, RBP/Cr, HbA1c, age, and hypertension. However, their study had a smaller patient sample size. In comparison, our study includes a larger patient population, and the predictive factors are easier to obtain in clinical practice.

SNPs are the most important genetic markers in the genome and continue to be a prominent focus in genetic research (21, 22). Many aspects of SNPs sites related to DKD remain unknown. Our study conducted SNPs genotyping analysis and identified new SNPs sites and genotypes associated with susceptibility to DKD within the gene functional region of AP5B1, a part of the adaptor protein complex 5 involved in endosomal transport (23). We analyzed that the rs6591190 may bind to transcription factor binding sites, thereby affecting gene expression. Exon missense mutation at rs12146493 may affect gene splicing, transcription or expression. Previous studies have shown that the rs4014195 locus of the AP5B1 gene is closely related to eGFR in both DM and non-DM patients (24). However, no correlation between the rs4014195 locus and DKD was found in our study, possibly due to the small sample size. As the primary focus of our study was on developing a clinical predictive model for DKD in T2DM patients with DR, the SNP analysis was conducted on an exploratory basis to identify potential genetic markers. Future studies with larger cohorts are needed to validate these findings.

### Limitations

Despite its strengths, our study has certain limitations. (1) DKD in our study was diagnosed based on clinical criteria without renal biopsy for pathological confirmation. (2) The study did not include all potential risk factors that may affect the occurrence of microvascular complications in T2DM patients, such as dietary habits, physical activity levels, education level, and other factors. (3) Due to the small sample size and various confounding factors in our study, whether these SNPs sites are susceptibility genotypes affecting DKD occurrence needs further validation through large-scale samples and genome-wide association study (GWAS). Additionally, animal models need to be constructed to explore the potential mechanisms of these SNPs sites in DKD pathogenesis. (4) Our study was retrospective and single-center, thus requiring more external data to validate the efficacy of the prediction model, particularly necessitating multicenter, large-sample prospective cohort studies to cover different regions.

## Conclusions

Our study includes easily accessible clinical and laboratory indicators and uses scientific statistical methods to construct a model predicting the risk of DKD in T2DM patients with DR. This model can guide clinical practice by controlling blood pressure, managing blood lipids, promoting weight loss, and improving nutrition, thereby preventing or delaying the onset of DKD in T2DM patients with DR. This, in turn, can reduce the economic burden on both patients and society. Additionally, the SNPs sites related to DKD identified in our study may provide new data support for this field. Future research should focus on expanding sample sizes, conducting multicenter studies, and exploring the underlying mechanisms of these genetic factors to further improve DKD risk prediction and prevention strategies.

## Data Availability

The original contributions presented in the study are included in the article/**Supplementary Material**, further inquiries can be directed to the corresponding author/s.
