# Supplementary figures and images for: Development and validation of a risk prediction model for diabetic kidney disease in patients with diabetic retinopathy
Source: Front Endocrinol (Lausanne). 2025 May 5;16:1499866. doi: 10.3389/fendo.2025.1499866 (PMC12086070; doi:10.3389/fendo.2025.1499866)

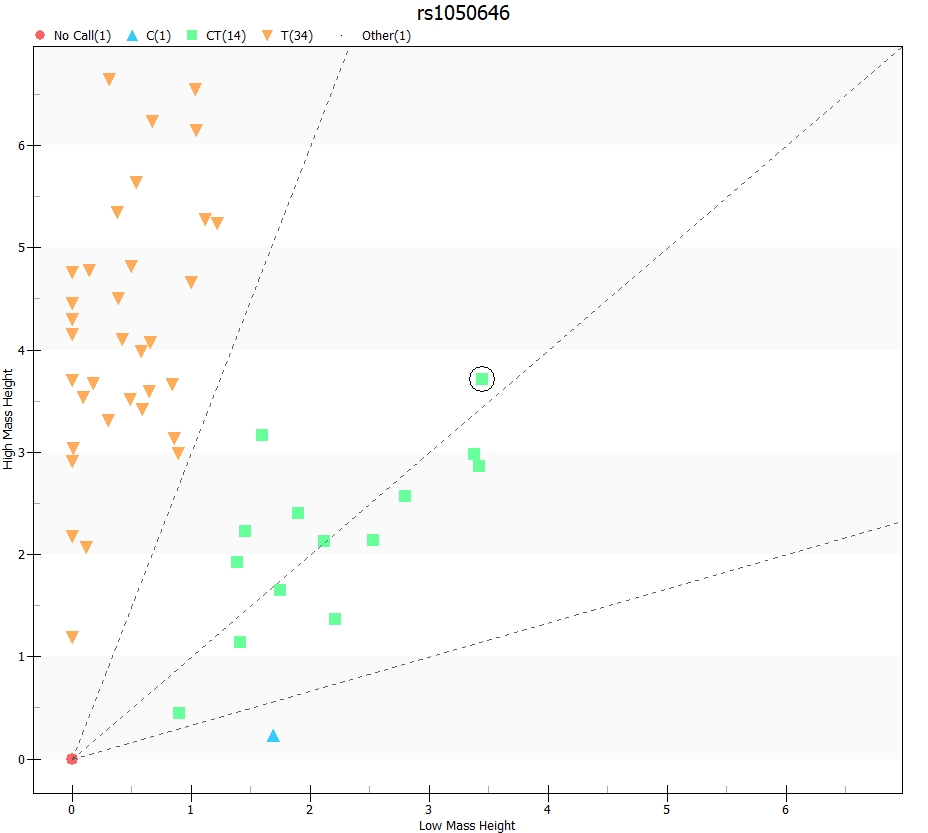

Supplement: Supplementary file 1 [file SupplementaryFile1.zip › original data/figure/rs1050646.jpeg]

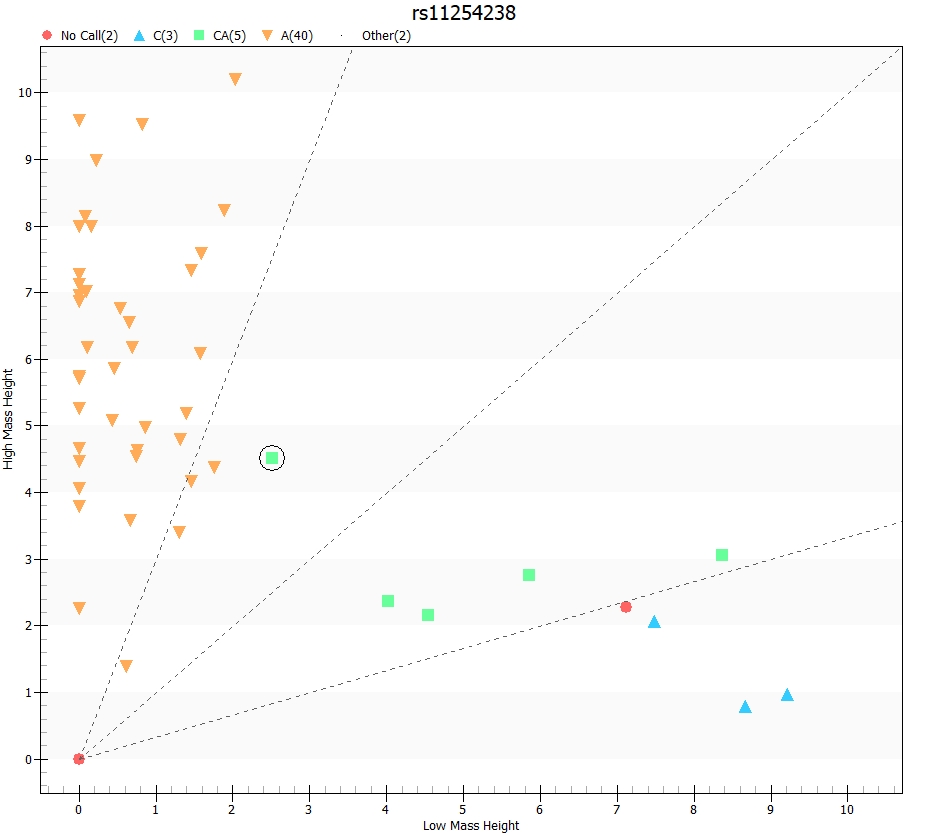

Supplement: Supplementary file 1 [file SupplementaryFile1.zip › original data/figure/rs11254238.jpeg]

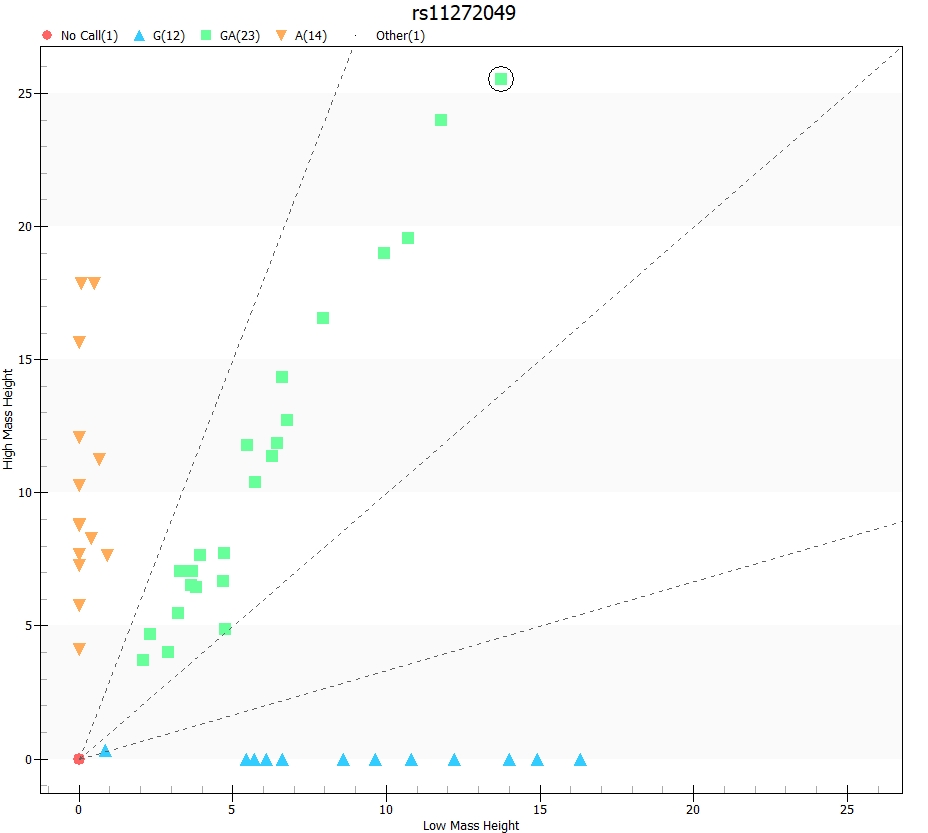

Supplement: Supplementary file 1 [file SupplementaryFile1.zip › original data/figure/rs11272049.jpeg]

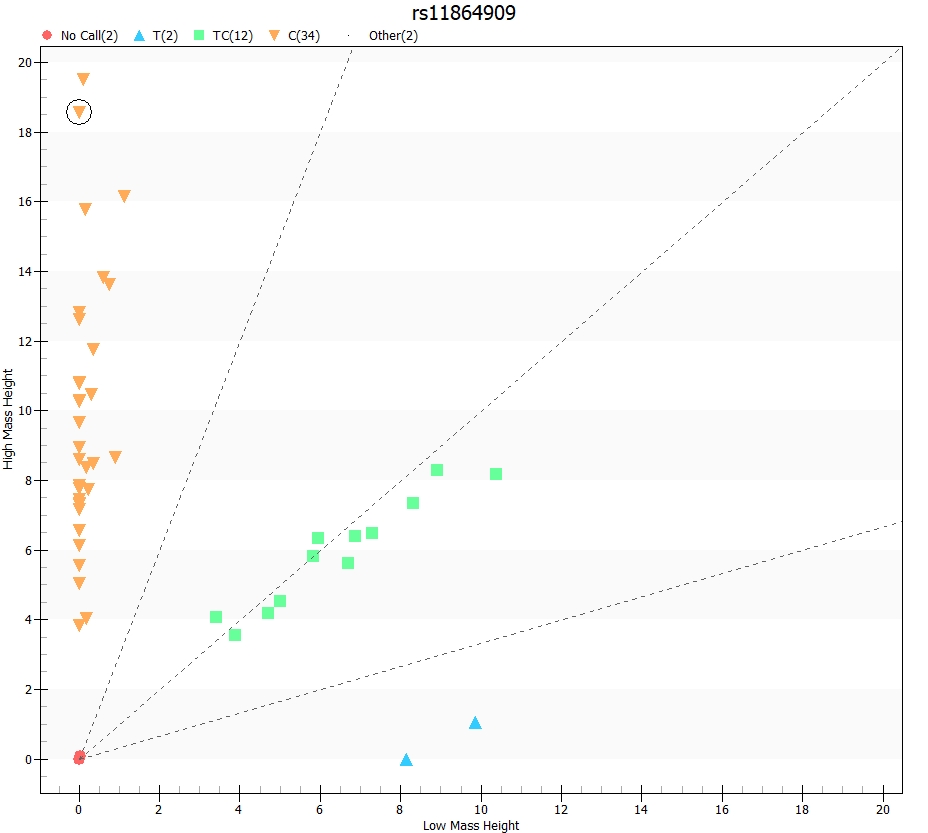

Supplement: Supplementary file 1 [file SupplementaryFile1.zip › original data/figure/rs11864909.jpeg]

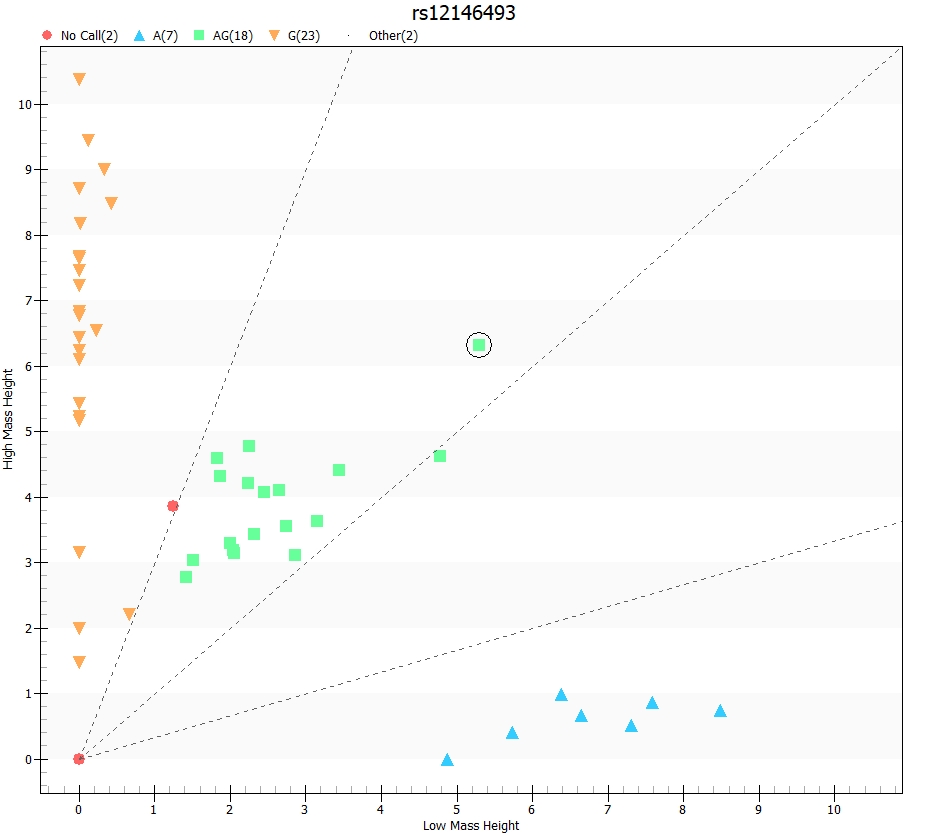

Supplement: Supplementary file 1 [file SupplementaryFile1.zip › original data/figure/rs12146493.jpeg]

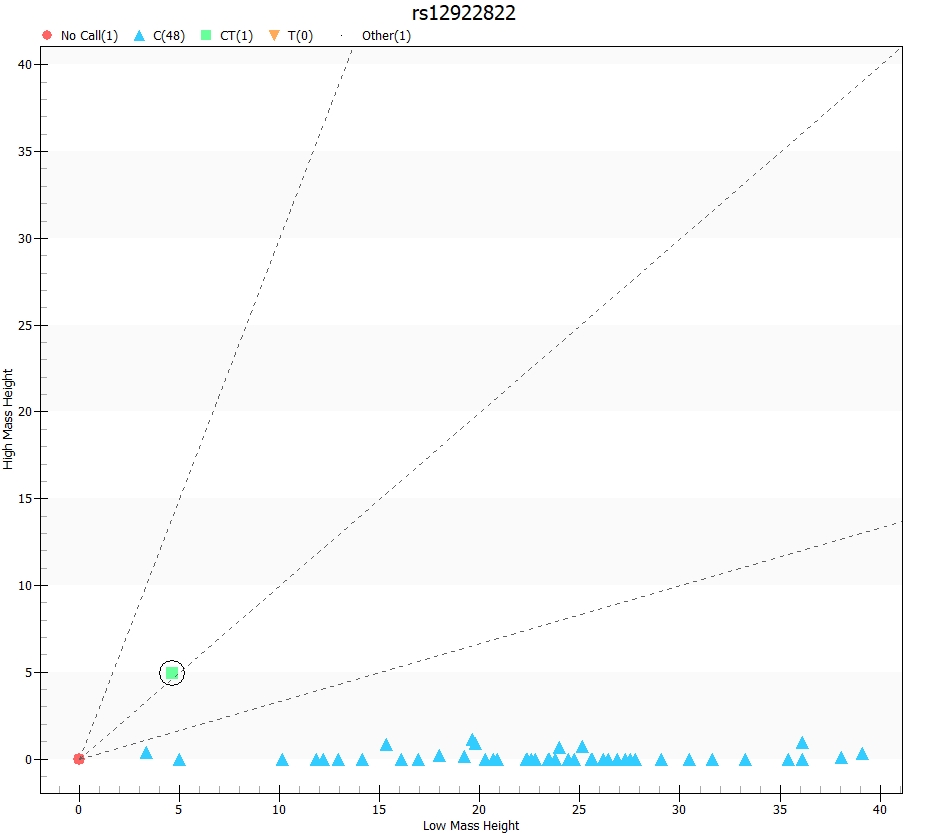

Supplement: Supplementary file 1 [file SupplementaryFile1.zip › original data/figure/rs12922822.jpeg]

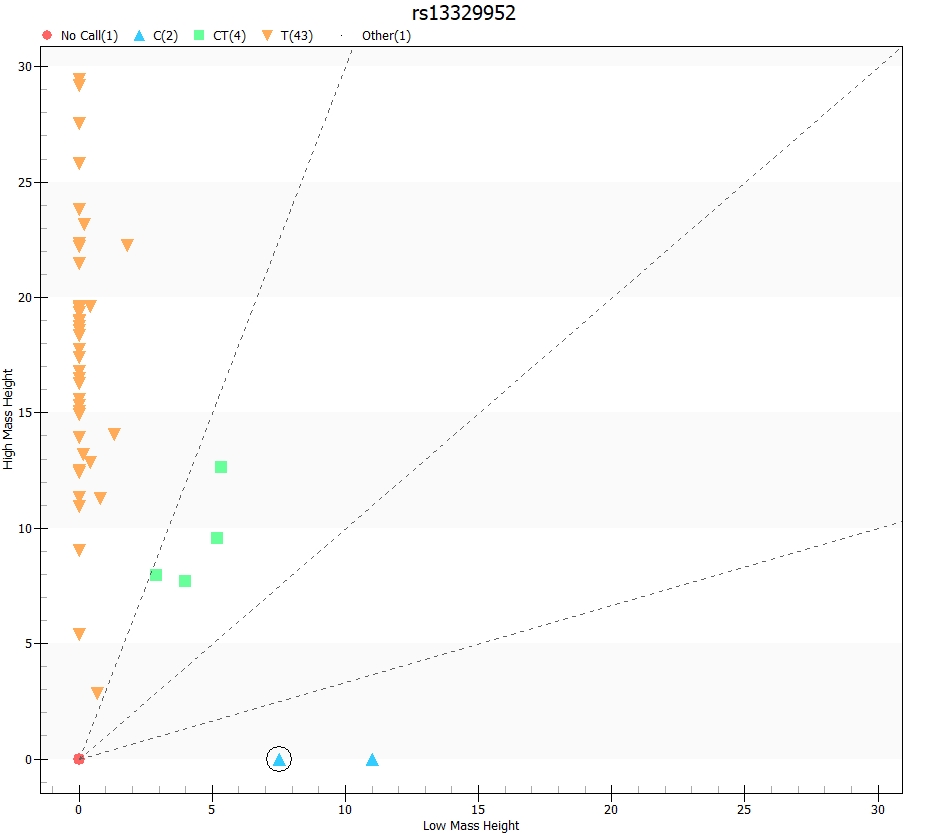

Supplement: Supplementary file 1 [file SupplementaryFile1.zip › original data/figure/rs13329952.jpeg]

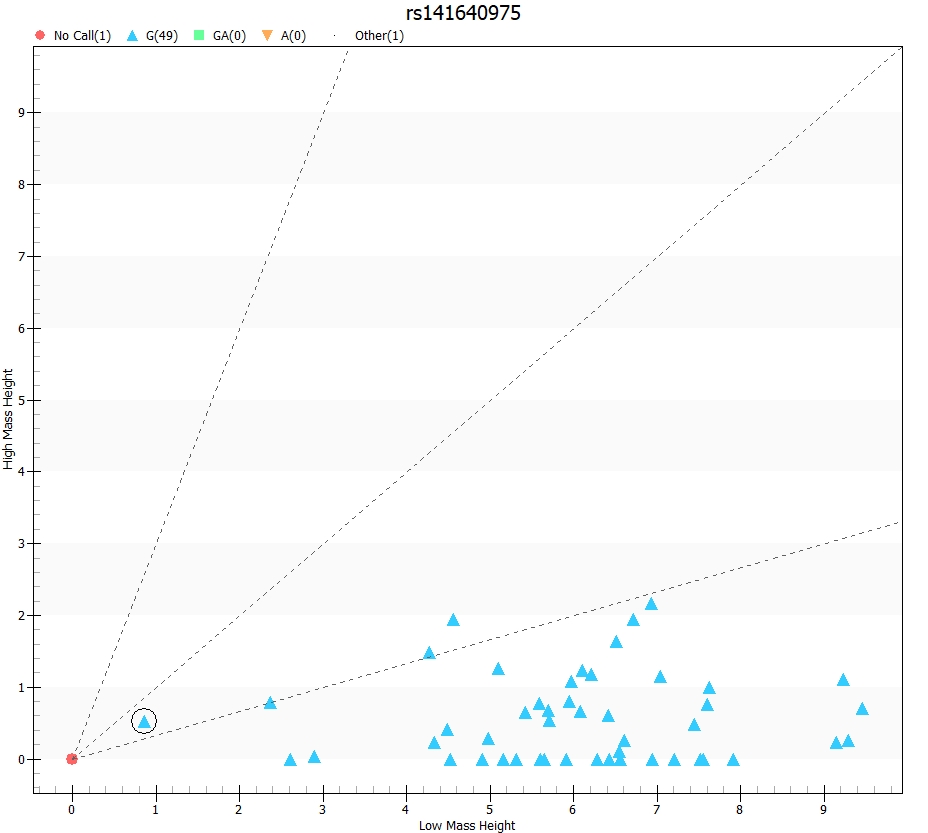

Supplement: Supplementary file 1 [file SupplementaryFile1.zip › original data/figure/rs141640975.jpeg]

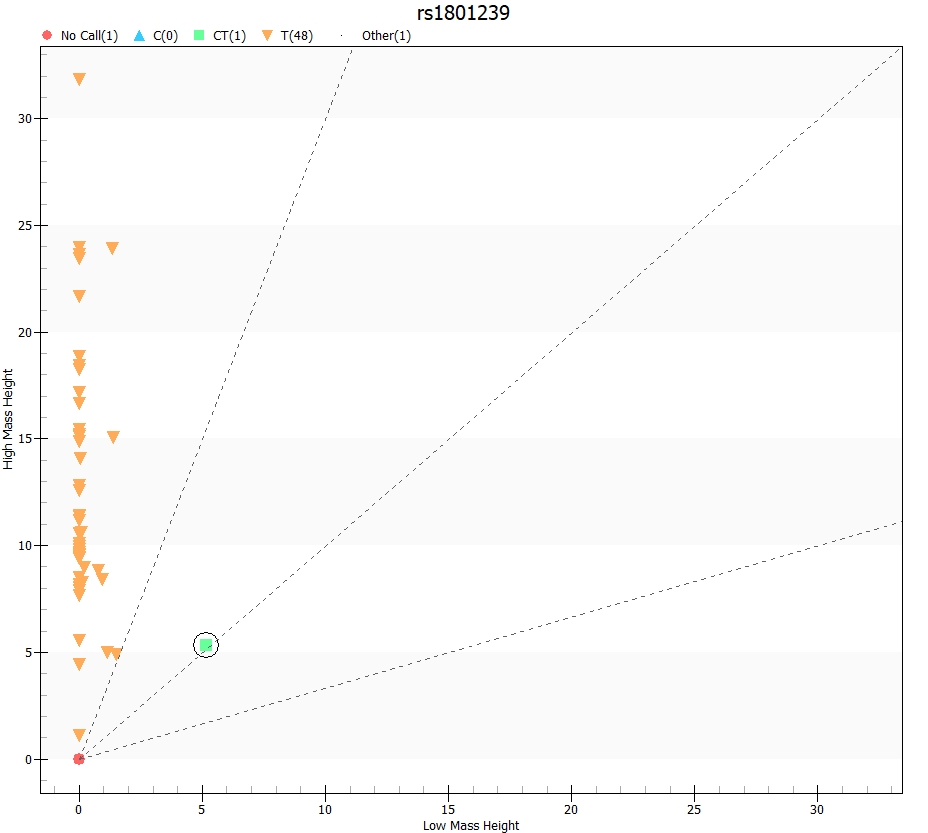

Supplement: Supplementary file 1 [file SupplementaryFile1.zip › original data/figure/rs1801239.jpeg]

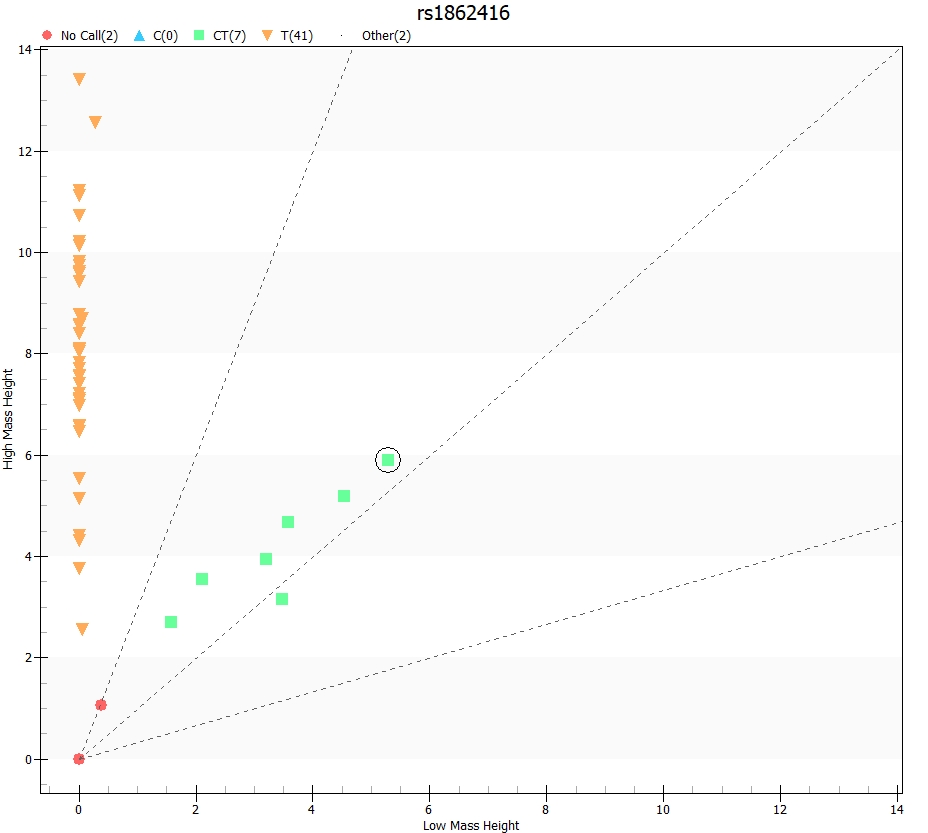

Supplement: Supplementary file 1 [file SupplementaryFile1.zip › original data/figure/rs1862416.jpeg]

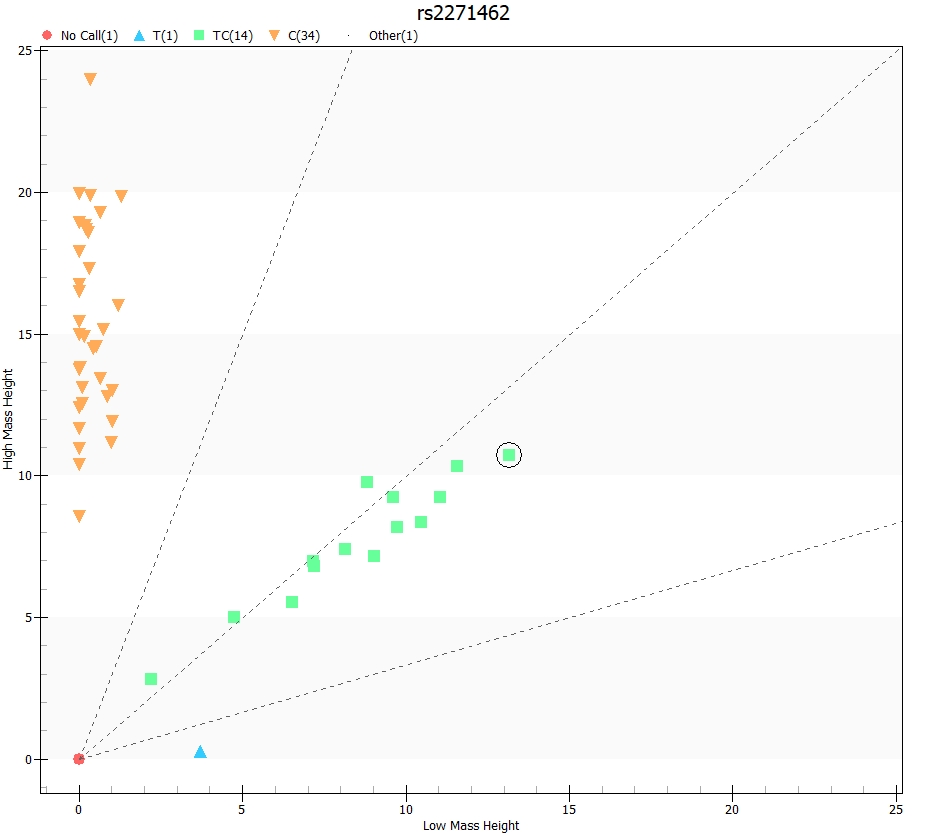

Supplement: Supplementary file 1 [file SupplementaryFile1.zip › original data/figure/rs2271462.jpeg]

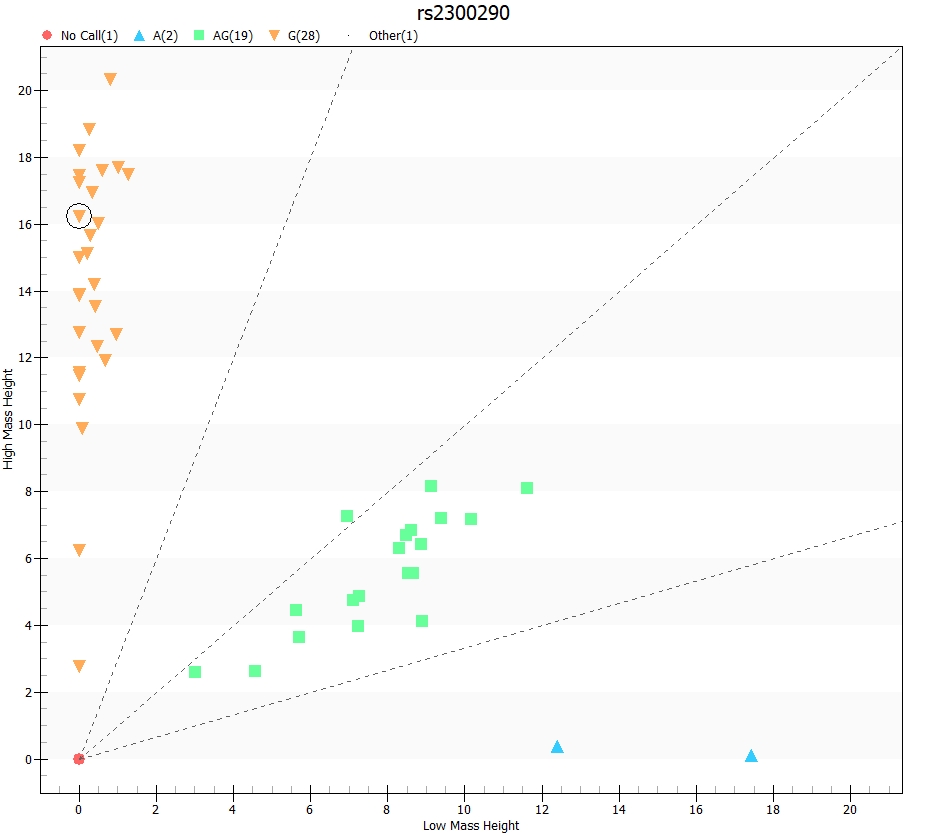

Supplement: Supplementary file 1 [file SupplementaryFile1.zip › original data/figure/rs2300290.jpeg]

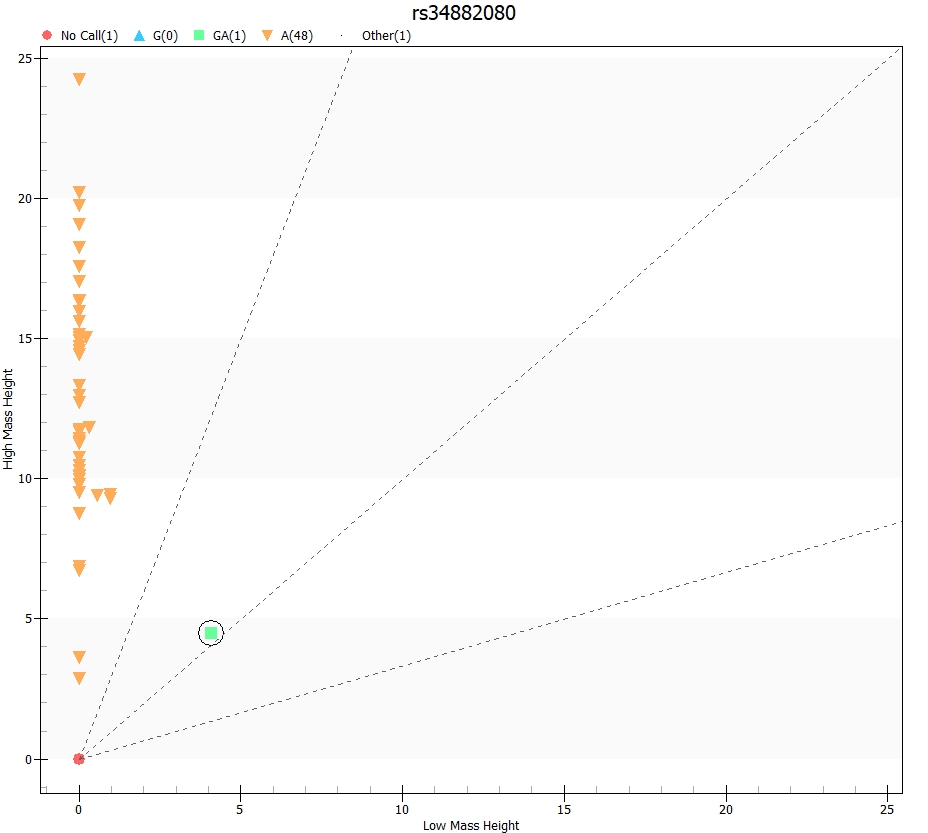

Supplement: Supplementary file 1 [file SupplementaryFile1.zip › original data/figure/rs34882080.jpeg]

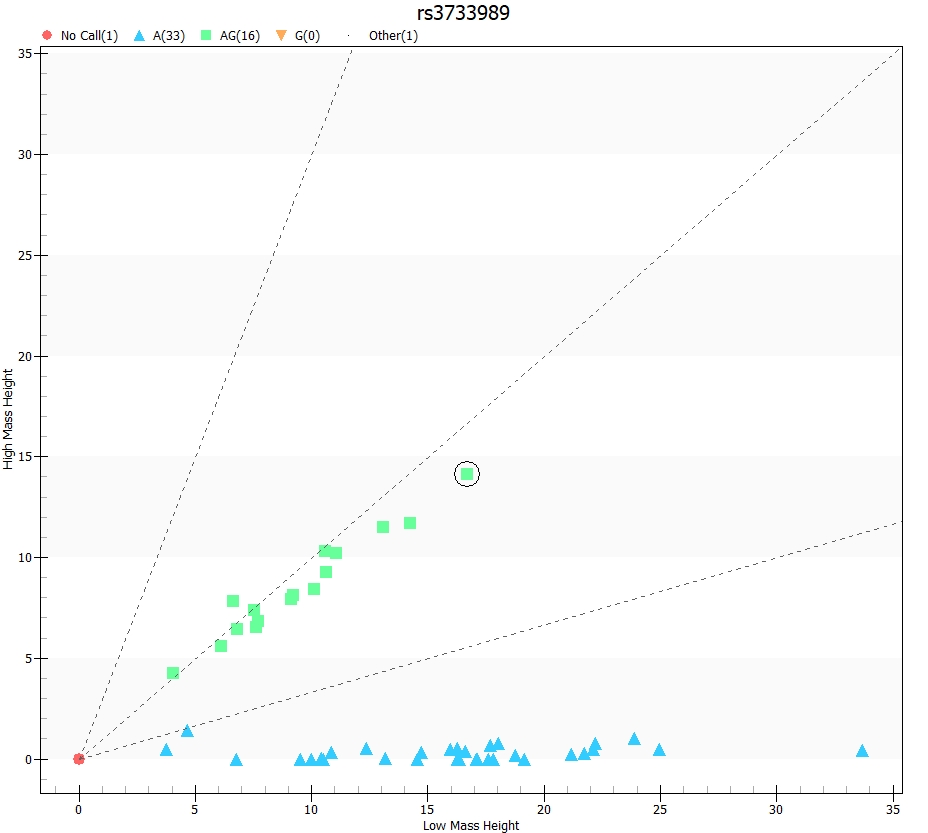

Supplement: Supplementary file 1 [file SupplementaryFile1.zip › original data/figure/rs3733989.jpeg]

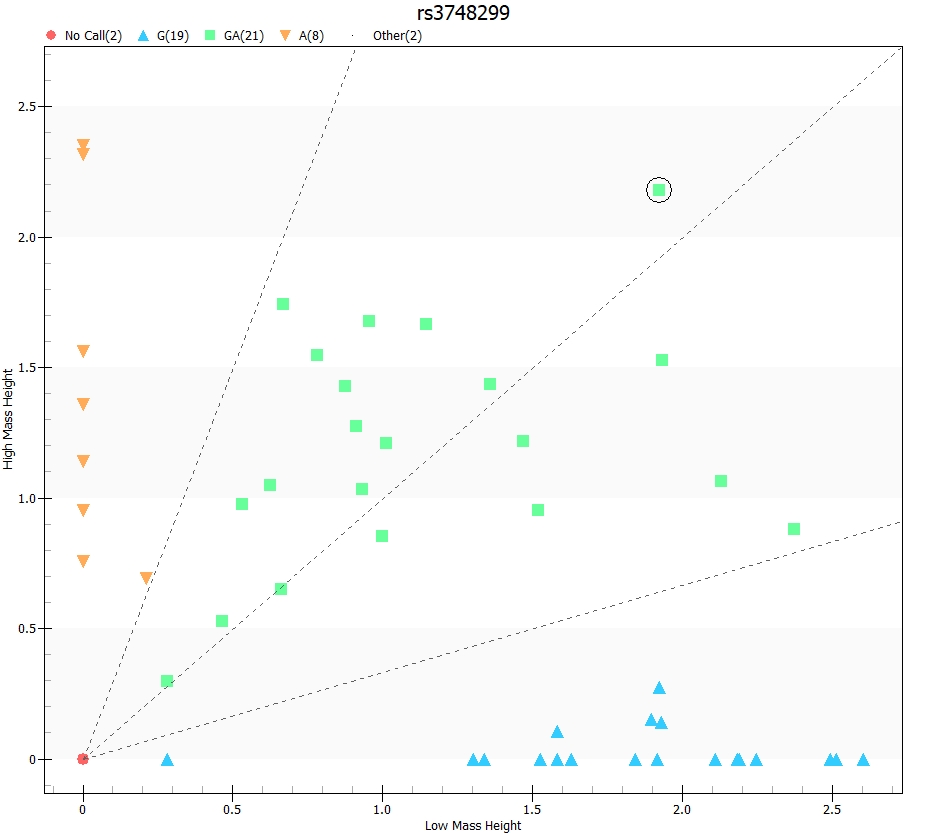

Supplement: Supplementary file 1 [file SupplementaryFile1.zip › original data/figure/rs3748299.jpeg]

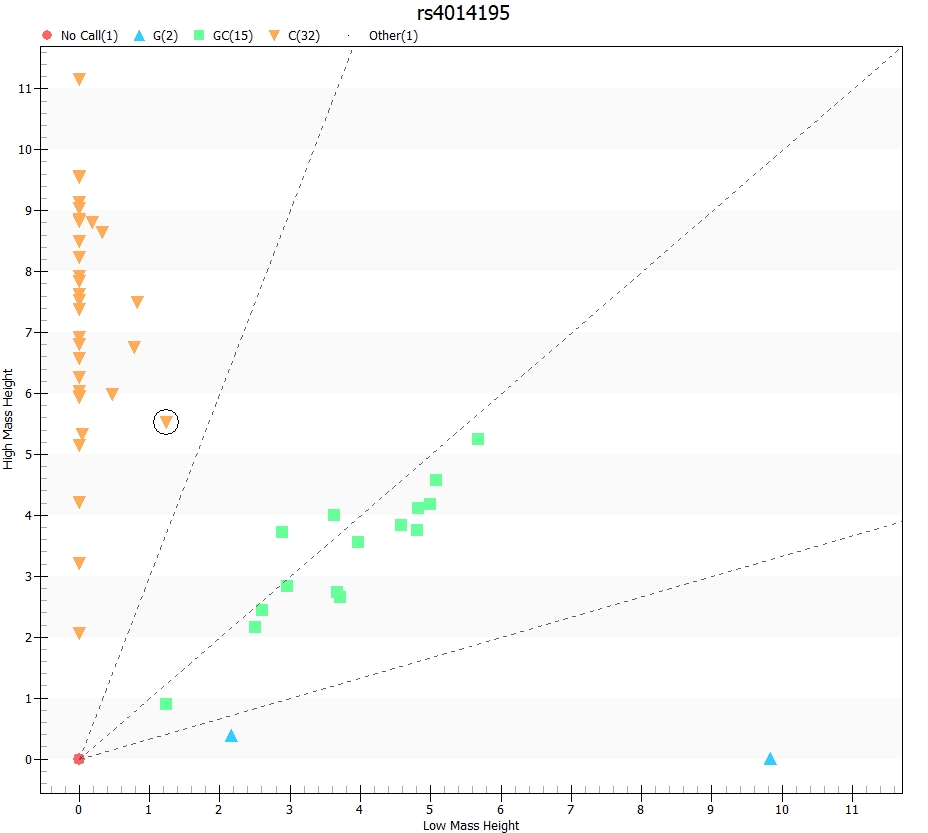

Supplement: Supplementary file 1 [file SupplementaryFile1.zip › original data/figure/rs4014195.jpeg]

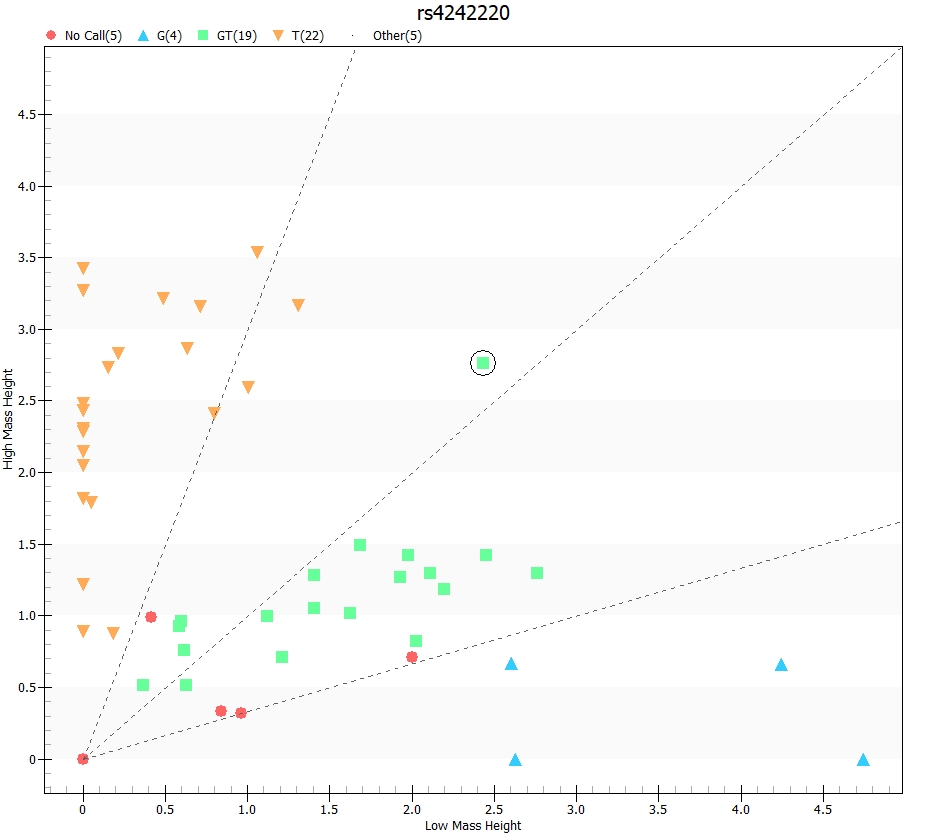

Supplement: Supplementary file 1 [file SupplementaryFile1.zip › original data/figure/rs4242220.jpeg]

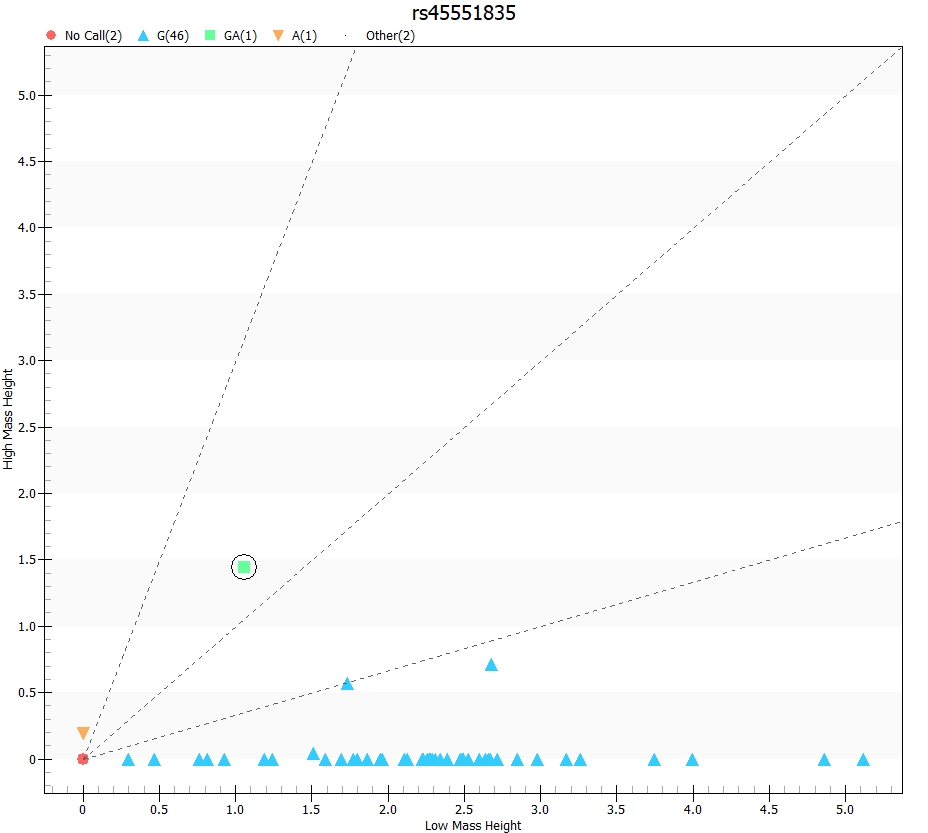

Supplement: Supplementary file 1 [file SupplementaryFile1.zip › original data/figure/rs45551835.jpeg]

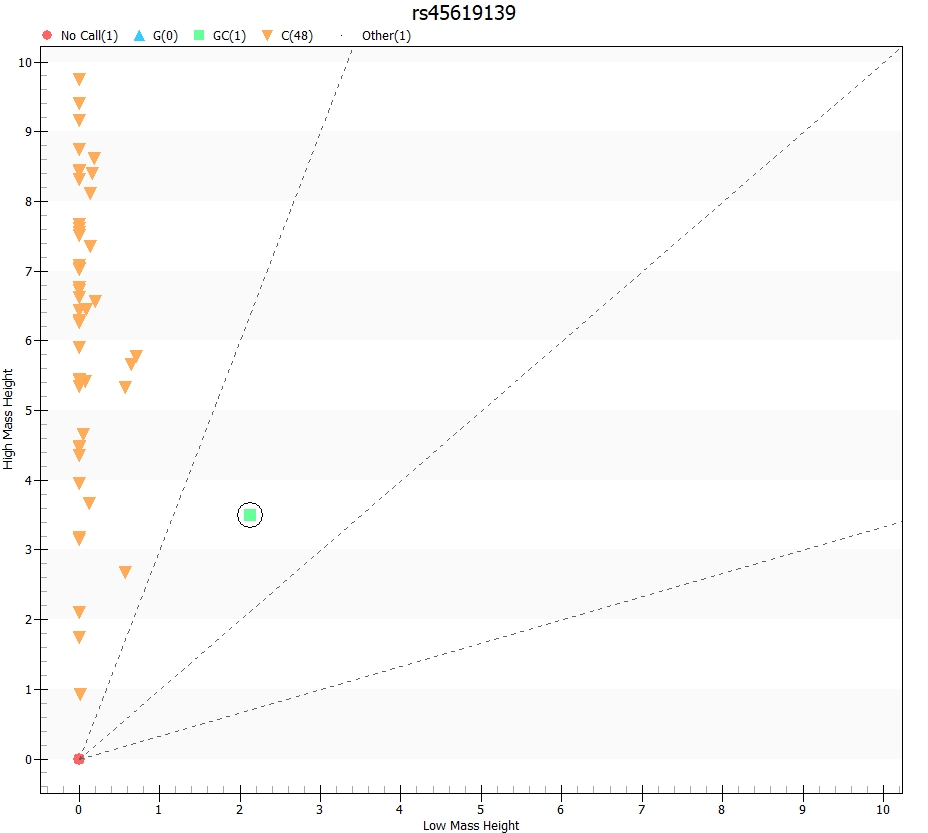

Supplement: Supplementary file 1 [file SupplementaryFile1.zip › original data/figure/rs45619139.jpeg]

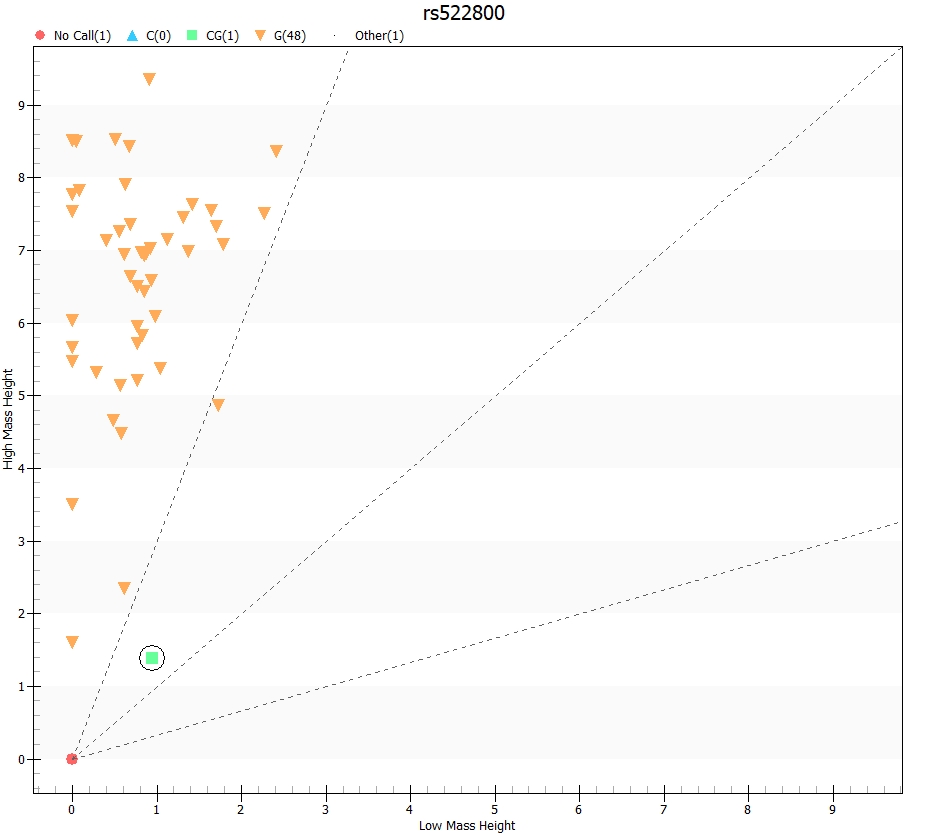

Supplement: Supplementary file 1 [file SupplementaryFile1.zip › original data/figure/rs522800.jpeg]

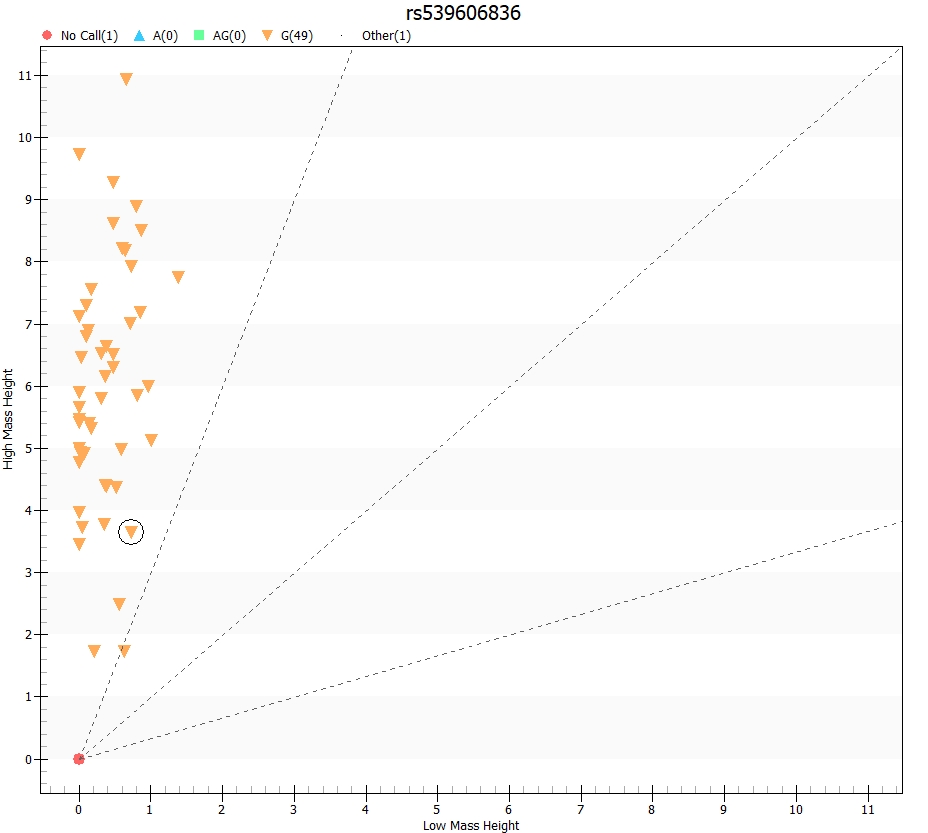

Supplement: Supplementary file 1 [file SupplementaryFile1.zip › original data/figure/rs539606836.jpeg]

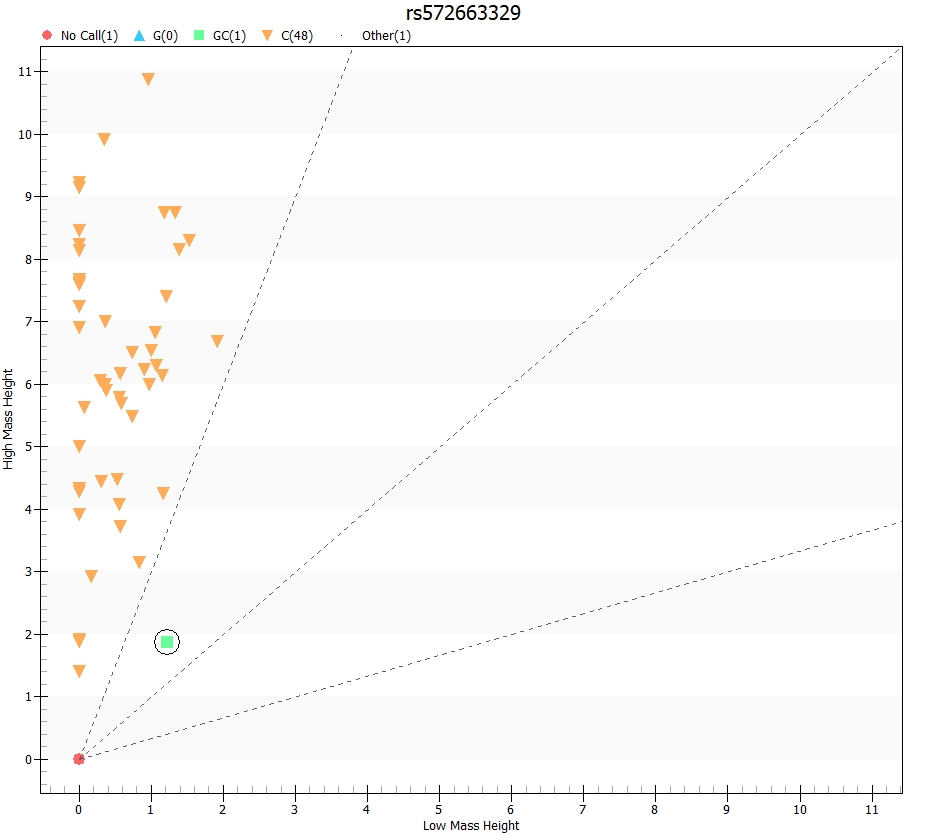

Supplement: Supplementary file 1 [file SupplementaryFile1.zip › original data/figure/rs572663329.jpeg]

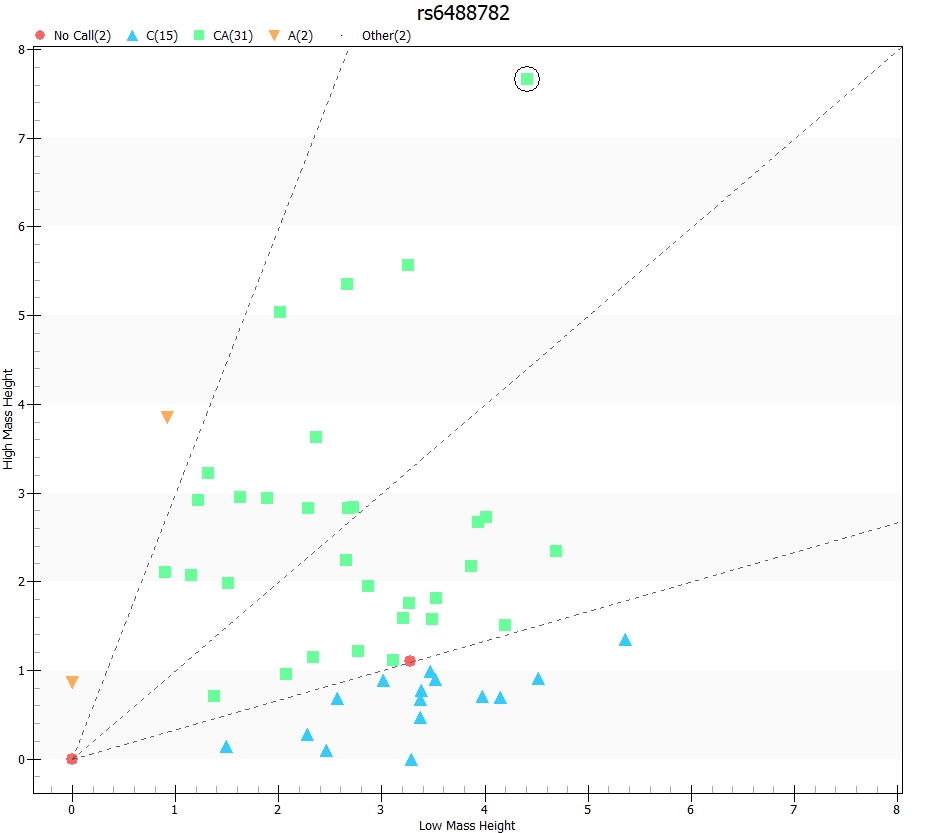

Supplement: Supplementary file 1 [file SupplementaryFile1.zip › original data/figure/rs6488782.jpeg]

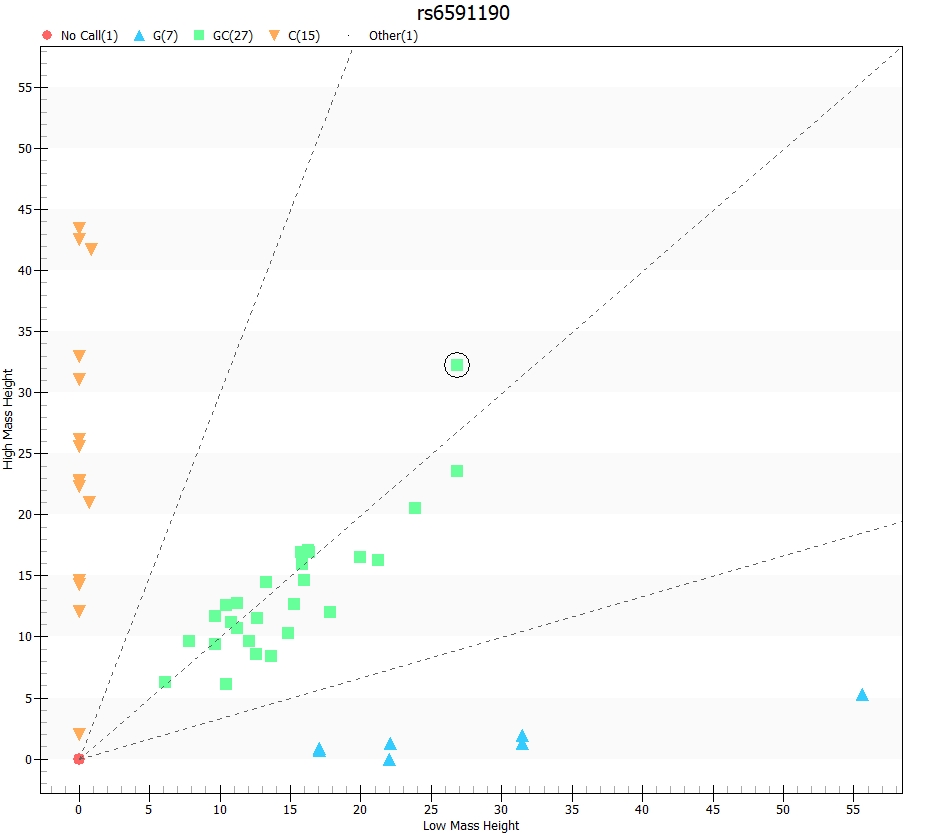

Supplement: Supplementary file 1 [file SupplementaryFile1.zip › original data/figure/rs6591190.jpeg]

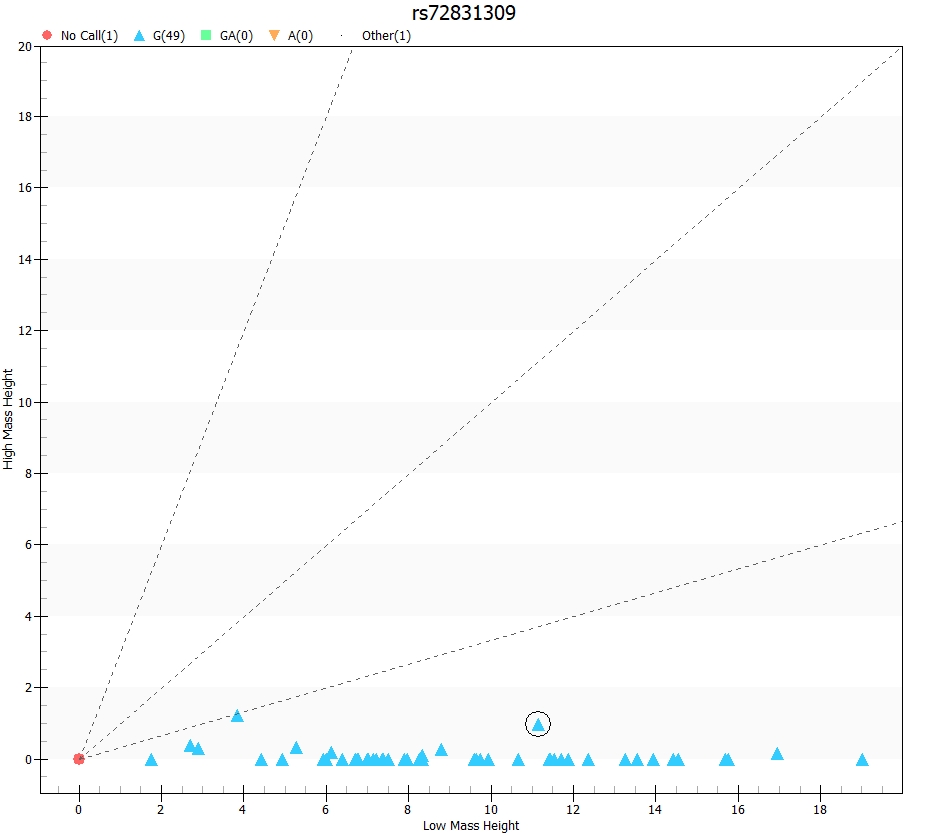

Supplement: Supplementary file 1 [file SupplementaryFile1.zip › original data/figure/rs72831309.jpeg]

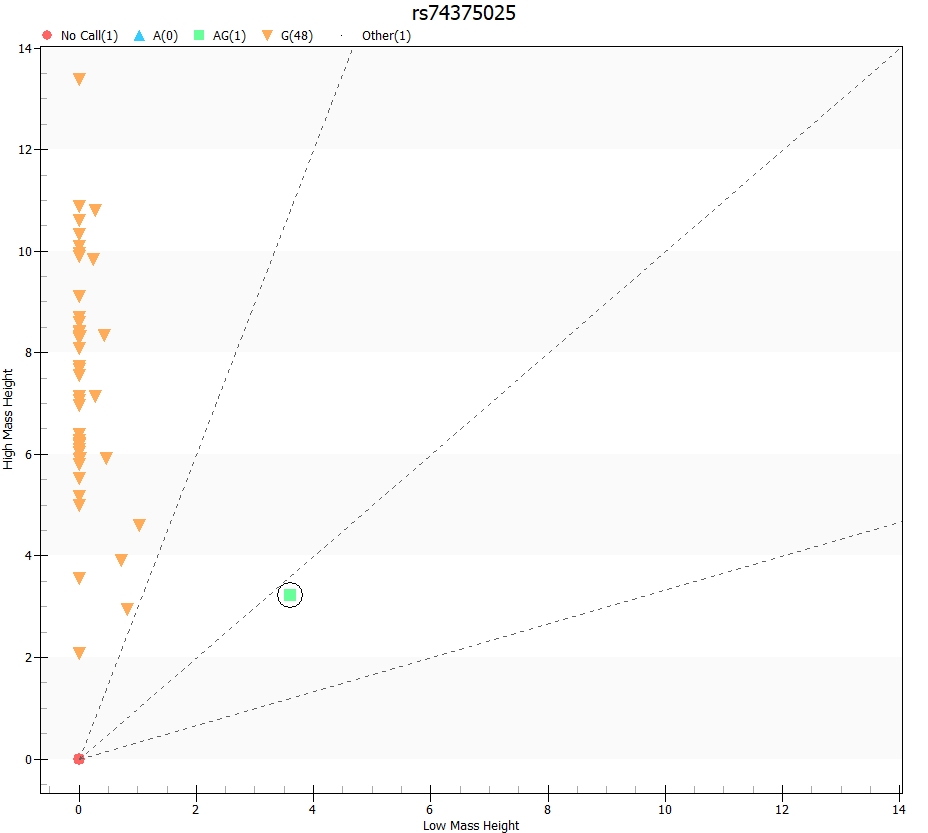

Supplement: Supplementary file 1 [file SupplementaryFile1.zip › original data/figure/rs74375025.jpeg]

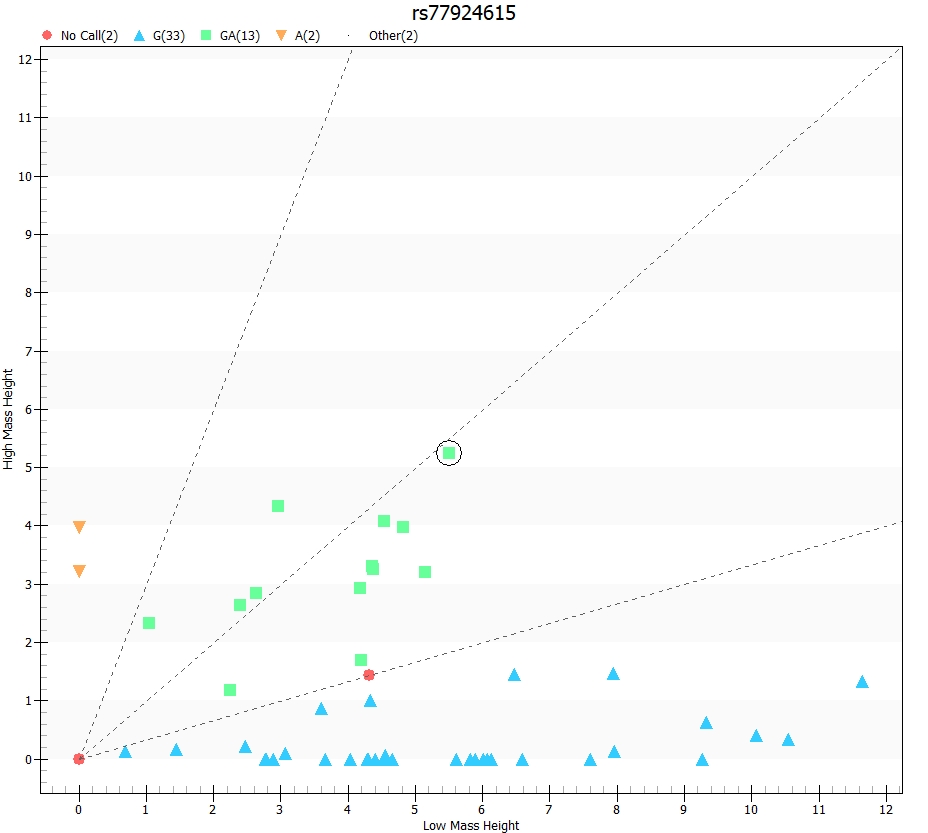

Supplement: Supplementary file 1 [file SupplementaryFile1.zip › original data/figure/rs77924615.jpeg]

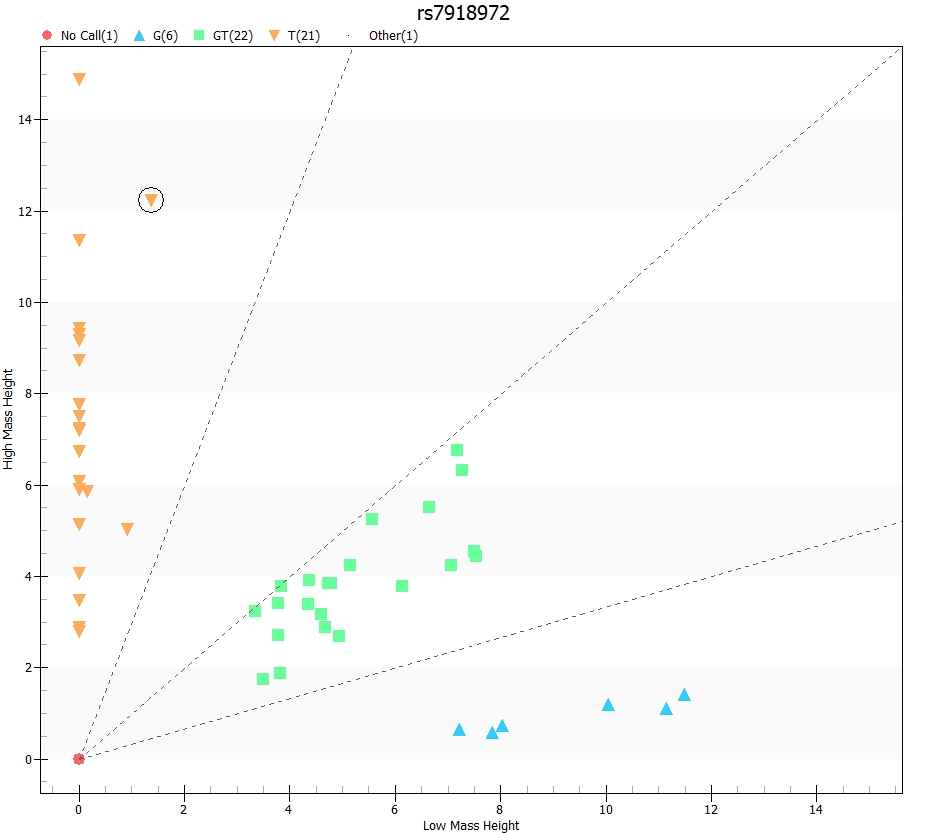

Supplement: Supplementary file 1 [file SupplementaryFile1.zip › original data/figure/rs7918972.jpeg]

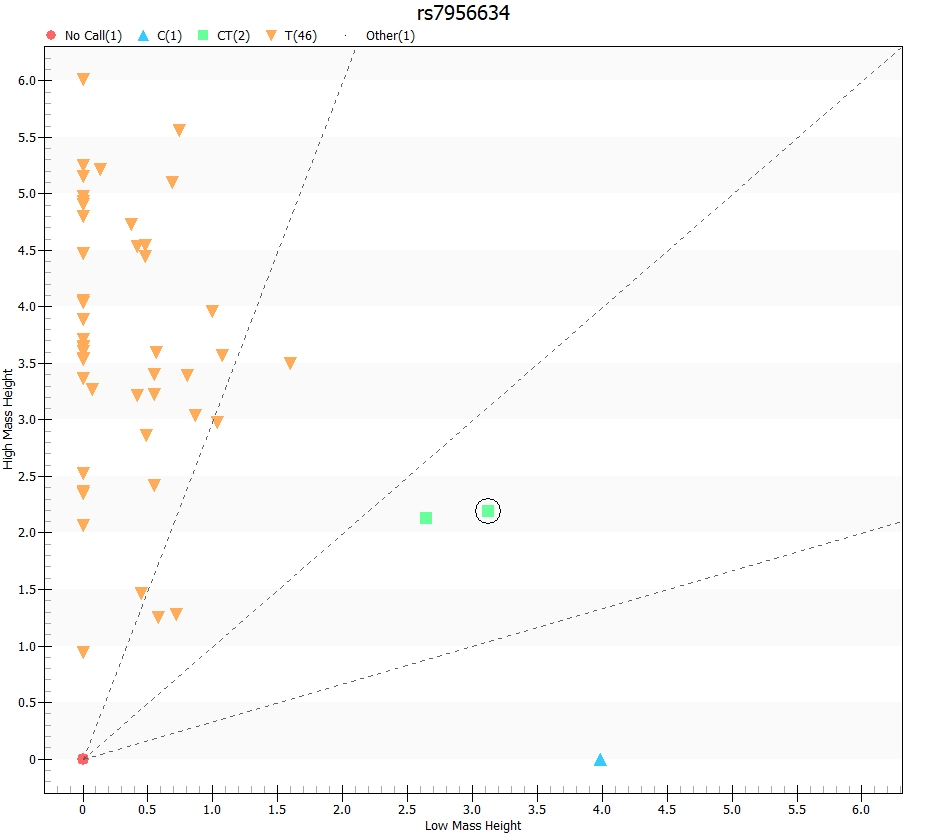

Supplement: Supplementary file 1 [file SupplementaryFile1.zip › original data/figure/rs7956634.jpeg]

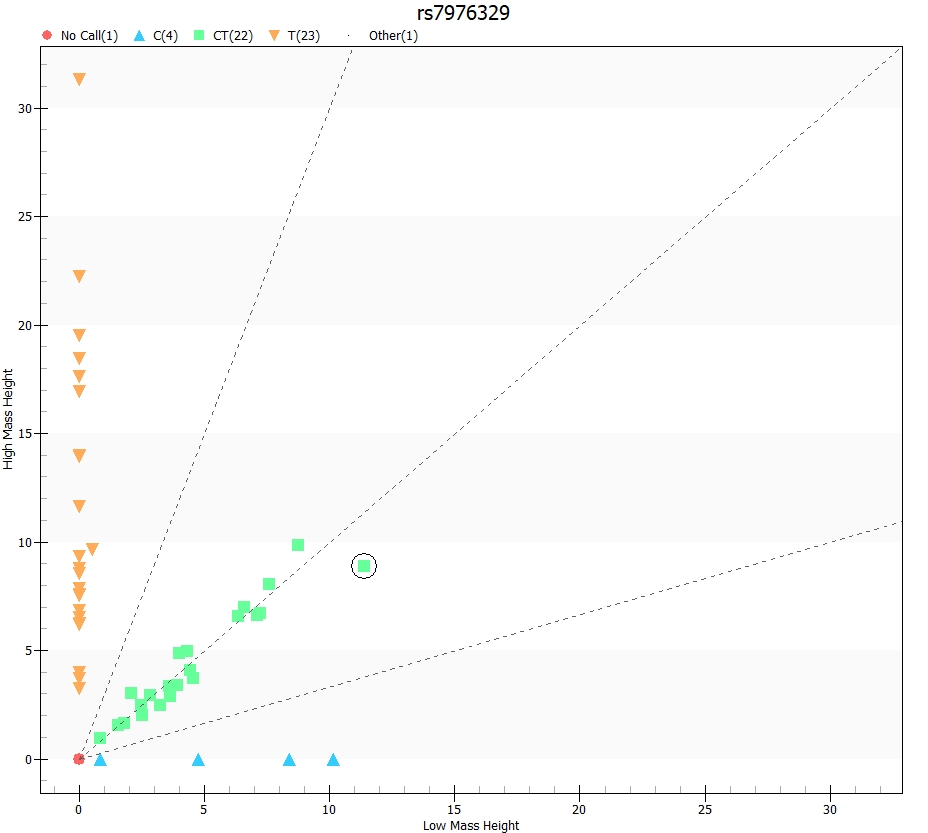

Supplement: Supplementary file 1 [file SupplementaryFile1.zip › original data/figure/rs7976329.jpeg]
