# Supplementary material for: Development and validation of a risk prediction model for diabetic kidney disease in patients with diabetic retinopathy
Source: Front Endocrinol (Lausanne). 2025 May 5;16:1499866. doi: 10.3389/fendo.2025.1499866 (PMC12086070; doi:10.3389/fendo.2025.1499866)
Supplement: Supplementary file 2 [file Table1.docx]

Supplementary Table 1. The main instruments and equipment

| Instrument Name | Manufacturer |
| --- | --- |
| Low-Speed Centrifuge | Eppendorf, German |
| QB-206 Shaker | Haimen Qilin Beier Instrument Manufacturing Co., Ltd. |
| Oscillator | Haimen Qilin Beier Instrument Manufacturing Co., Ltd. |
| 0.5-10μl、20-200μl、100-1000μl Tip | Axygen Scientific Inc |
| Pipette | Eppendorf, German |
| NanoDrop2000 | Thermo Fisher Scientific |
| Automatic Nucleic Acid Extractor | Nanjing Zhongke Bayer |
| MP-300V Electrophoresis Instrument | Major Science（MS） |
| Smart View Pro 1100 Gel Electrophoresis Imaging System | Major Science（MS） |
| ABI Veriti-384 PCR System | Applied Biosystems |
| MassARRAY Analyzer 4.0 Mass Spectrometer | Agena Bioscience, Inc |
| 384-well SpectroCHIP® Bioarray | Agena Bioscience, Inc |
| MassARRAY Nanodispenser | Agena Bioscience, Inc |

Supplementary Table 2. The main experimental reagents and consumables

| Reagent Name | Manufacturer |
| --- | --- |
| DNA Extraction Kit | Nanjing Zhongke Bayer |
| Agarose | HydraGene Co.,Ltd. |
| DNA Stain | Biomed Corpration |
| 50×TAE Buffer | Biomed Corpration |
| 6×Loading Buffer | Biomed Corpration |
| Primer Synthesis | Thermo Fisher Scientific Inc. |
| 10×PCR Buffer | Agena Bioscience, Inc |
| 25 mM dNTP Mix | Agena Bioscience, Inc |
| 15mM and 25mM MgCl2 | Agena Bioscience, Inc |
| SAP Buffer | Agena Bioscience, Inc |
| SAP Enzyme | Agena Bioscience, Inc |
| HotStar Taq(5U/μl) | Agena Bioscience, Inc |
| iPLEX Buffer Plus | Agena Bioscience, Inc |
| iPLEX Termination mix | Agena Bioscience, Inc |
| iPLEX Enzyme | Agena Bioscience, Inc |
| MassARRAY TYPER 4.0 | Agena Bioscience, Inc |

Supplementary Table 3. Baseline characteristics of training and validation sets

| Characteristic | Training set (N=478) | Validation set (N=205) | | t/Z/χ² | | *P* value |
| --- | --- | --- | --- | --- | --- | --- |
| Group (DR group) | 207（43.31%） | 86（41.95%） | | 0.107 | | 0.743 |
| Age (y) | 56.00（49.00,62.00） | 55.00（48.00,61.00） | | 1.211 | | 0.226 |
| Gender (male) | 325（67.99%） | 138（67.32%） | | 0.030 | | 0.863 |
| Classification of DR  (NPDR) | 407（85.15%） | 178（86.83%） | | 0.331 | | 0.565 |
| Age at T2DM  diagnosis (y) | 42.00（36.00,50.00） | 40.00（33.00,49.00） | | 1.410 | | 0.159 |
| Duration of T2DM (m) | 132.00(72.00,228.00) | 144.00(92.00,228.00) | | 0.651 | | 0.515 |
| History of  hypertension (yes) | 275（57.53%） | 121（59.02%） | | 0.131 | | 0.717 |
| Duration of  hypertension (m) | 12.00（0,120.00） | 12.00（0,107.00） | | 0.031 | | 0.976 |
| History of coronary  heart disease (yes) | 100（20.92%） | 36（17.56%） | | 1.015 | | 0.314 |
| History of stroke (yes) | 103（21.55%） | 35（17.07%） | | 1.782 | | 0.182 |
| History of fatty liver  (yes) | 307（64.23%） | 121（59.02%） | | 1.659 | | 0.198 |
| Smoking history (yes) | 155（32.43%） | 72（35.12%） | | 0.470 | | 0.493 |
| Years of smoking (y) | 0（0,18.00） | 0（0,20.00） | | 0.952 | | 0.425 |
| Alcohol consumption  history (yes) | 106（22.18%） | 34（16.59%） | | 2.751 | | 0.097 |
| Years of alcohol  consumption (y) | 0（0,3.00） | 0（0,3.00） | | 0.347 | | 0.786 |
| Family history of  T2DM (yes) | 193（40.38%） | 86（41.95%） | | 0.147 | | 0.701 |
| Systolic blood  pressure (mmHg) | 134.50(125.00,146.00) | 135.00(125.00,150.00) | | 1.086 | | 0.278 |
| Diastolic blood  pressure (mmHg) | 80.00（76.00,88.00） | 80.00（77.00,87.00） | | 0.094 | | 0.926 |
| BMI (kg/m^2^) | 26.10（23.89,28.08） | 26.20（24.10,29.00） | | 1.548 | | 0.122 |
| 24h urinary microalbumin(mg)  DR group  DR combined DKD group | 10.8（6.92,15.19）  308.8（78.66,1489.31） | 10.8（5.00,14.29）  234.04（99.13,974.49） | | 0.949  0.588 | | 0.342  0.556 |
| 24h urinary albumin (g) | 0.23（0.13,0.72） | 0.24（0.12,0.70） | | 0.562 | | 0.574 |
| Urine specific gravity | 1.02（1.01,1.03） | 1.02（1.01,1.03） | | 0.737 | | 0.462 |
| Urine PH | 5.50（5.50,6.50） | 5.50（5.50,6.50） | | 0.182 | | 0.859 |
| BUN (mmol/L) | 6.08（4.87,7.46） | 6.04（5.14,7.42） | | 0.522 | | 0.602 |
| Cr (umol/L) | 69.45（57.50,87.97） | 71.40（56.70,86.10） | | 0.160 | | 0.873 |
| UA (umol/L) | 351.00（293.00,409.00） | 358.00（299.00,429.00） | | 0.867 | | 0.386 |
| RBP (mg/L) | 47.55（37.97,58.48） | 49.80（39.50,60.10） | 1.404 | | 0.160 | |
| Cys-C (mg/L) | 0.93（0.80,1.12） | 0.93（0.79,1.10） | 0.245 | | 0.807 | |
| eGFR (ml/min)  DR group  DR combined DKD group | 100.83（93.13,109.84）  91.06（64.28,104.68） | 100.6（92.93,107.85）  89.49（70.06,103.52） | 0.201  0.251 | | 0.841  0.802 | |
| WBC (10^9^/L) | 6.33（5.34,7.61） | 6.30（5.26,7.36） | 0.299 | | 0.765 | |
| NE (10^9^/L) | 3.67（2.99,4.5） | 3.61（2.93,4.55） | 0.064 | | 0.949 | |
| LY (10^9^/L) | 2.00（1.61,2.43） | 2.01（1.59,2.39） | 0.211 | | 0.833 | |
| MO (10^9^/L) | 0.42（0.32,0.52） | 0.43（0.33,0.53） | 0.830 | | 0.407 | |
| RBC (10^12^/L) | 4.67（4.25,5.10） | 4.74（4.32,5.16） | 1.045 | | 0.296 | |
| HGB (g/L) | 141.50（127.00,154.00） | 143.00（130.00,155.00） | 1.222 | | 0.222 | |
| HCT (L/L) | 0.41（0.37,0.45） | 0.42（0.39,0.45） | 1.302 | | 0.193 | |
| RDW (%) | 12.60（12.10,13.00） | 12.60（12.10,13.00） | 0.348 | | 0.728 | |
| PLT (10^9^/L) | 211.50（174.25,250.00） | 209.00（177.00,250.00） | 0.143 | | 0.886 | |
| PCT (%) | 0.22（0.19,0.26） | 0.22（0.19,0.26） | 0.058 | | 0.954 | |
| APTT (s) | 26.55（24.8,29.20） | 26.60（24.90,29.10） | 0.445 | | 0.656 | |
| PT (s) | 10.90（10.40,11.40） | 11.00（10.50,11.50） | 1.873 | | 0.065 | |
| FIB (g/L) | 2.96（2.58,3.62） | 2.99（2.52,3.57） | 0.206 | | 0.837 | |
| AST (U/L) | 19.40（16.20,24.90） | 19.90（16.40,23.80） | 0.090 | | 0.928 | |
| ALT (U/L) | 19.50（13.30,28.50） | 20.30（14.40,28.60） | 0.538 | | 0.591 | |
| γ-GT (U/L) | 28.00（18.70,45.98） | 28.60（20.30,40.90） | 0.017 | | 0.986 | |
| ALP (U/L) | 73.00（60.42,88.20） | 70.70（58.70,89.90） | 0.890 | | 0.373 | |
| ALB (g/L) | 38.95（36,41.08） | 38.80（35.80,41.30） | 0.383 | | 0.702 | |
| TBil (umol/L) | 12.10（9.22,15.00） | 12.10（9.60,16.10） | 0.678 | | 0.498 | |
| DBil (umol/L) | 2.40（1.70,3.00） | 2.30（1.70,3.20） | 0.168 | | 0.867 | |
| TC (mmol/L) | 4.78（4.10,5.75） | 5.01（4.26,5.83） | 1.382 | | 0.167 | |
| TG (mmol/L) | 1.83（1.26,2.98） | 2.07（1.35,3.03） | 1.120 | | 0.263 | |
| HDL-C (mmol/L) | 1.06（0.89,1.27） | 1.06（0.92,1.24） | 0.191 | | 0.848 | |
| LDL-C (mmol/L) | 2.98（2.32,3.66） | 3.06（2.47,3.69） | 1.385 | | 0.166 | |
| Potassium (mmol/L) | 3.96（3.71,4.17） | 3.92（3.68,4.22） | 0.184 | | 0.854 | |
| Sodium (mmol/L) | 140.90（138.80,142.67） | 141.10（139.00,143.00） | 0.935 | | 0.350 | |
| Chloride (mmol/L) | 105.00（103.12,106.90） | 105.00（102.70,106.80） | 0.754 | | 0.451 | |
| Calcium (mmol/L) | 2.19（2.13,2.27） | 2.20（2.13,2.28） | 0.541 | | 0.589 | |
| Phosphorus (mmol/L) | 1.19（1.07,1.30） | 1.20（1.09,1.36） | 1.022 | | 0.307 | |
| FBG (mmol/L) | 7.97（6.37,10.03） | 8.05（6.50,10.03） | 0.192 | | 0.847 | |
| HbA1c (%) | 8.80（7.70,10.00） | 8.80（7.60,10.30） | 0.391 | | 0.696 | |
| TSH (uIU/ml) | 1.92（1,22,2.74） | 1.77（1.19,2.80） | 0.274 | | 0.784 | |
| FT3 (mmol/L) | 4.19（3.67,4.63） | 4.17（3.73,4.68） | 0.144 | | 0.886 | |
| FT4 (mmol/L) | 13.46（12.12,15.49） | 13.46（12.13,15.07） | 0.499 | | 0.618 | |

Abbreviations: DR, diabetic retinopathy; NPDR, non-proliferative diabetic retinopathy; T2DM, type 2 diabetes mellitus; BMI, body mass index; DKD, diabetic kidney disease; BUN, blood urea nitrogen; Cr, creatinine; UA, uric acid; RBP, retinol binding protein; Cys-C, cystatin C; eGFR, estimated glomerular filtration rate; WBC, white blood cell; NE, neutrophil absolute value; LY, lymphocyte absolute value; MO, monocyte absolute value; RBC, red blood cell; HGB, hemoglobin; HCT, hematocrit value; RDW, red blood cell distribution width; PLT, blood platelet; PCT, platelet hematocrit; APTT, activated partial thromboplastin time; PT, prothrombin time; FIB, fibrinogen; AST, aspartate aminotransferase; ALT, alanine aminotransferase; γ-GT, γ-glutamyl transpeptidase; ALP, alkaline phosphatase; ALB, albumin; TBil, total bilirubin; DBil, direct bilirubin; TC, total cholesterol; TG, triglyceride; HDL-C, high-density lipoprotein cholesterol; LDL-C, low density lipoprotein cholesterol; FBG, fasting blood-glucose; HbA1c, glycosylated hemoglobin; TSH, thyroid stimulating hormone; FT3, free triiodothyronine; FT4, free thyroxine.

Supplementary Table 4. Univariate logistic regression analysis of training set

| Variables | Beta | S.E. | Z | OR (95%CI) | *P* value |
| --- | --- | --- | --- | --- | --- |
| Gender (female vs. male) | -0.85 | 0.2 | -4.26 | 0.43（0.29-0.63） | ＜0.001* |
| Age (y) | -0.01 | 0.01 | -1.15 | 0.99（0.97-1.01） | 0.249 |
| Classification of DR  (PDR vs. NPDR) | 0.85 | 0.29 | 2.99 | 2.35（1.34-4.11） | 0.003* |
| Age at T2DM diagnosis (y) | -0.01 | 0.01 | -1.39 | 0.99（0.97-1.01） | 0.166 |
| Duration of T2DM (m) | 0.00 | 0.00 | 0.29 | 1.00（1.00-1.00） | 0.768 |
| History of hypertension  (no vs. yes) | -0.88 | 0.19 | -4.64 | 0.41（0.28-0.60） | ＜0.001* |
| Duration of hypertension (m) | 0.00 | 0.00 | 1.14 | 1.00（1.00-1.00） | 0.254 |
| History of coronary heart disease (yes vs. no) | 0.02 | 0.23 | 0.07 | 1.02(1.65-1.59) | 0.945 |
| History of stroke (yes vs. no) | 0.55 | 0.23 | 2.36 | 1.74(1.10-2.75) | 0.018* |
| History of fatty liver  (no vs. yes) | -0.14 | 0.19 | -0.73 | 0.87(0.60-1.26) | 0.468 |
| Smoking history (yes vs. no) | 0.60 | 0.20 | 2.97 | 1.83（1.23-2.72） | 0.003* |
| Years of smoking (y) | 0.02 | 0.01 | 2.50 | 1.02（1.01-1.03） | 0.012* |
| Alcohol consumption history  (yes vs. no) | 0.14 | 0.22 | 0.64 | 1.16（0.75-1.79） | 0.519 |
| Years of alcohol  consumption (y) | 0.04 | 0.02 | 1.21 | 1.03（0.97-1.10） | 0.213 |
| Family history of T2DM  (yes vs. no) | 0.02 | 0.19 | 0.11 | 1.02（0.71-1.48） | 0.913 |
| Systolic blood pressure (mmHg) | 0.03 | 0.01 | 5.65 | 1.04（1.02-1.05） | ＜0.001* |
| Diastolic blood pressure (mmHg) | 0.02 | 0.01 | 1.64 | 1.02（1.00-1.03） | 0.101 |
| BMI (kg/m^2^) | 0.12 | 0.03 | 4.06 | 1.13（1.06-1.19） | ＜0.001* |
| APTT (s) | 0.02 | 0.03 | 0.71 | 1.02（0.97-1.07） | 0.479 |
| PT (s) | -0.11 | 0.12 | -0.96 | 0.89（0.71-1.13） | 0.338 |
| FIB (g/L) | 0.63 | 0.12 | 5.11 | 1.88（1.48-2.40） | ＜0.001* |
| ALT (U/L) | -0.01 | 0.01 | -1.75 | 0.99（0.98-1.00） | 0.080 |
| AST (U/L) | -0.01 | 0.01 | -1.98 | 0.98（0.96-1.01） | 0.068 |
| γ-GT (U/L) | 0.00 | 0.00 | 0.78 | 1.00（1.00-1.01） | 0.433 |
| ALP (U/L) | 0.00 | 0.00 | 1.05 | 1.00（1.00-1.01） | 0.295 |
| ALB (g/L) | -0.12 | 0.02 | -5.3 | 0.89（0.85-0.93） | ＜0.001* |
| TBil (umol/L) | -0.03 | 0.02 | -1.76 | 0.97（0.94-1.00） | 0.079 |
| TC (mmol/L) | 0.18 | 0.07 | 2.6 | 1.20（1.04-1.37） | 0.009* |
| TG (mmol/L) | 0.06 | 0.04 | 1.72 | 1.07（0.99-1.14） | 0.086 |
| HDL-C (mmol/L) | -0.77 | 0.28 | -2.78 | 0.46（0.27-0.80） | 0.005* |
| LDL-C (mmol/L) | 0.27 | 0.10 | 2.68 | 1.31（1.08-1.60） | 0.007* |
| Residual cholesterol (mmol/L) | 0.69 | 0.19 | 3.55 | 1.98（1.36-1.89） | ＜0.001* |
| AI | 0.46 | 0.09 | 5.38 | 1.59（1.34-1.89） | ＜0.001* |
| API | 0.49 | 0.09 | 5.38 | 1.59（1.34-1.89） | ＜0.001* |
| AIP | 1.46 | 0.32 | 4.54 | 4.33（2.30-8.14） | ＜0.001* |
| FBG (mmol/L) | 0.04 | 0.03 | 1.26 | 1.04（0.98-1.11） | 0.208 |
| TyG index | 0.54 | 0.12 | 4.38 | 1.71（1.34-2.17） | ＜0.001* |
| HbA1c (%) | 0.00 | 0.05 | 0.10 | 1.00（0.91-1.10） | 0.923 |
| TSH (uIU/ml) | -0.07 | 0.05 | -1.41 | 0.94（0.85-1.03） | 0.159 |
| FT3 (mmol/L) | -0.33 | 0.12 | -2.71 | 0.72（0.56-0.91） | 0.007* |
| FT4 (mmol/L) | -0.03 | 0.03 | -0.94 | 0.97（0.92-1.03） | 0.347 |

Abbreviations: S.E., Standard error; NPDR, non-proliferative diabetic retinopathy; PDR, proliferative diabetic retinopathy; T2DM, type 2 diabetes mellitus; BMI, body mass index; APTT, activated partial thromboplastin time; PT, prothrombin time; FIB, fibrinogen; ALT, alanine aminotransferase; AST, aspartate aminotransferase; γ-GT, γ-glutamyl transpeptidase; ALP, alkaline phosphatase; ALB, albumin; TBil, total bilirubin; TC, total cholesterol; TG, triglyceride; HDL-C, high-density lipoprotein cholesterol; LDL-C, low density lipoprotein cholesterol; AI, atherogenic index of plasma; API, atherogenic plasma index; AIP, atherogenic index of plasma; FBG, fasting blood-glucose; HbA1c, glycosylated hemoglobin; TSH, thyroid stimulating hormone; FT3, free triiodothyronine; FT4, free thyroxine. *: P < 0.05, with statistical difference.

Supplementary Table 5. The results of SNPs genotyping

| Gene | SNPs locus | Allele | MAF | *HWP* Value (Overall) | *HWP* Value  (DR group) | *HWP* Value  (DR with DKD group) |
| --- | --- | --- | --- | --- | --- | --- |
| AP5B1 | rs4014195 | G/C | 0.1939 | 1 | 0.6145 | 1 |
|  | rs6591190 | G/C | 0.4184 | 0.5558 | 1 | 0.0802 |
|  | rs522800 | C/G | 0.0102 | 1 | 1 | 1 |
|  | rs12146493 | A/G | 0.3333 | 0.3295 | 0.4374 | 1 |
| TENM2 | rs3733989 | G/A | 0.1633 | 0.3259 | 1 | 1 |
|  | rs1862416 | C/T | 0.0729 | 1 | 1 | 1 |
|  | rs4242220 | G/T | 0.3000 | 1 | 0.6253 | 0.3497 |
|  | rs11272049 | G/A | 0.4796 | 0.7747 | 0.7103 | 0.2198 |
| CUBN | rs11254238 | C/A | 0.1146 | 1 | 0.4097 | 0.1781 |
|  | rs74375025 | A/G | 0.0102 | 1 | 1 | 1 |
|  | rs7918972 | G/T | 0.3469 | 1 | 0.4097 | 0.1781 |
|  | rs1801239 | C/T | 0.0102 | 1 | 1 | 1 |
|  | rs45619139 | G/C | 0.0102 | 1 | 1 | 1 |
|  | rs45551835 | A/G | 0.0313 | 0.0316 | 1 | 0.0243 |
|  | rs572663329 | G/C | 0.0102 | 1 | 1 | 1 |
|  | rs2271462 | T/C | 0.1633 | 1 | 1 | 1 |
| UMOD | rs13329952 | C/T | 0.0816 | 0.0218 | 0.0056 | 1 |
|  | rs11864909 | T/C | 0.1667 | 0.5943 | 0.5718 | 1 |
|  | rs77924615 | A/G | 0.1771 | 0.6207 | 0.5783 | 1 |
|  | rs34882080 | G/A | 0.0102 | 1 | 1 | 1 |
|  | rs12922822 | T/C | 0.0102 | 1 | 1 | 1 |
| PTPRO | rs7976329 | C/T | 0.3061 | 1 | 1 | 1 |
|  | rs2300290 | A/G | 0.2347 | 1 | 0.2885 | 0.5683 |
|  | rs3748299 | A/G | 0.3854 | 0.5579 | 1 | 0.6527 |
|  | rs1050646 | C/T | 0.1633 | 1 | 0.5488 | 1 |
|  | rs7956634 | C/T | 0.0408 | 0.0615 | 1 | 0.1378 |
|  | rs6488782 | A/C | 0.3646 | 0.0113 | 0.0376 | 0.3376 |

Abbreviations: MAF, minor allele frequency; HWP, Hardy-Weinberg P.

Supplementary Table 6. Genotype logistic regression analysis of the remaining SNPs loci

| Gene | SNPs locus | genetic model | genotype | OR (95%CI) | *P* value |
| --- | --- | --- | --- | --- | --- |
| AP5B1 | rs4014195 | Codominant | C/C  G/C  G/G | 1  0.67 (0.19-2.31)  0.00 (0.00-NA) | 0.240 |
|  |  | Dominant | C/C  G/C-G/G | 1  0.55 (0.16-1.83) | 0.320 |
|  |  | Recessive | C/C-G/C  G/G | 1  0.00 (0.00-NA) | 0.120 |
|  |  | Over-dominant | C/C-G/G  G/C | 1  0.75 (0.22-2.57) | 0.650 |
|  |  | Log-additive | C/C  G/G | 1  0.50 (0.17-1.47) | 0.190 |
| TENM2 | rs4242220 | Codominant | T/T  G/T  G/G | 1  0.70 (0.20-2.45)  3.60 (0.32-30.23) | 0.370 |
|  |  | Dominant | T/T  G/T-G/G | 1  0.92 (0.28-2.99) | 0.890 |
|  |  | Recessive | T/T-G/T  G/G | 1  4.24 (0.41-34.27) | 0.190 |
|  |  | Over-dominant | T/T-G/G  G/T | 1  0.58 (0.17-1.95) | 0.380 |
|  |  | Log-additive | T/T  G/G | 1  1.24 (0.50-3.08) | 0.640 |
| TENM2 | rs11272049 | Codominant | A/A  G/A  G/G | 1  0.40 (0.10-1.56)  0.75 (0.16-3.53) | 0.380 |
|  |  | Dominant | A/A  G/A-G/G | 1  0.50 (0.14-1.76) | 0.280 |
|  |  | Recessive | A/A-G/A  G/G | 1  1.31 (0.36-4.84) | 0.680 |
|  |  | Over-dominant | A/A-G/G  G/A | 1  0.46 (0.14-1.45) | 0.180 |
|  |  | Log-additive | A/A  G/G | 1  0.84 (0.39-1.83) | 0.660 |
| CUBN | rs11254238 | Codominant | A/A  C/A  C/C | 1  1.83 (0.28-12.19)  0.61 (0.05-7.30) | 0.740 |
|  |  | Dominant | A/A  C/A-C/C | 1  1.22 (0.27-5.59) | 0.800 |
|  |  | Recessive | A/A-C/A  C/C | 1  0.57 (0.05-6.76) | 0.650 |
|  |  | Over-dominant | A/A-C/C  C/A | 1  1.89 (0.29-12.51) | 0.500 |
|  |  | Log-additive | A/A  C/C | 1  0.99 (0.35-2.78) | 0.980 |
| CUBN | rs7918972 | Codominant | T/T  G/T  G/G | 1  0.42 (0.12-1.47)  1.82 (0.27-12.17) | 0.200 |
|  |  | Dominant | T/T  G/T-G/G | 1  0.59 (0.19-1.85) | 0.360 |
|  |  | Recessive | T/T-G/T  G/G | 1  2.78 (0.46-16.84) | 0.250 |
|  |  | Over-dominant | T/T-G/G  G/T | 1  0.37 (0.12-1.21) | 0.094 |
|  |  | Log-additive | T/T  G/G | 1  0.95 (0.41-2.19) | 0.910 |
| CUBN | rs45551835 | Codominant | G/G  G/A  A/A | 1  0.00 (0.00-NA)  NA (0.00-NA) | 0.250 |
|  |  | Dominant | G/G  G/A-A/A | 1  1.30 (0.08-22.08) | 0.860 |
|  |  | Recessive | G/G-G/A  A/A | 1  NA (0.00-NA) | 0.190 |
|  |  | Over-dominant | G/G-A/A  G/A | 1  0.00 (0.00-NA) | 0.280 |
|  |  | Log-additive | G/G  A/A | 1  1.81 (0.26-12.54) | 0.530 |
| CUBN | rs2271462 | Codominant | C/C  T/C  T/T | 1  1.43 (0.41-4.99)  NA (0.00-NA) | 0.380 |
|  |  | Dominant | C/C  T/C-T/T | 1  1.63 (0.48-5.55) | 0.430 |
|  |  | Recessive | C/C-T/C  T/T | 1  NA (0.00-NA) | 0.200 |
|  |  | Over-dominant | C/C-T/T  T/C | 1  1.33 (0.38-4.62) | 0.650 |
|  |  | Log-additive | C/C  T/T | 1  1.78 (0.58-5.47) | 0.310 |
| UMOD | rs13329952 | Codominant | T/T  C/T  C/C | 1  3.79 (0.36-39.41)  0.00 (0.00-NA) | 0.140 |
|  |  | Dominant | T/T  C/T-C/C | 1  1.26 (0.23-6.98) | 0.790 |
|  |  | Recessive | T/T-C/T  C/C | 1  0.00 (0.00-NA) | 0.120 |
|  |  | Over-dominant | T/T-C/C  C/T | 1  4.11 (0.40-42.59) | 0.200 |
|  |  | Log-additive | T/T  C/C | 1  0.79 (0.23-2.75) | 0.710 |
| UMOD | rs11864909 | Codominant | C/C  T/C  T/T | 1  0.50 (0.13-1.98)  0.00 (0.00-NA) | 0.180 |
|  |  | Dominant | C/C  T/C-T/T | 1  0.40 (0.10-1.53) | 0.170 |
|  |  | Recessive | C/C-T/C  T/T | 1  0.00 (0.00-NA) | 0.120 |
|  |  | Over-dominant | C/C-T/T  T/C | 1  0.56 (0.14-2.19) | 0.400 |
|  |  | Log-additive | C/C  T/T | 1  0.39 (0.12-1.31) | 0.100 |
| UMOD | rs77924615 | Codominant | G/G  G/A  A/A | 1  0.59 (0.16-2.18)  0.00 (0.00-NA) | 0.210 |
|  |  | Dominant | G/G  G/A-A/A | 1  0.47 (0.13-1.68) | 0.240 |
|  |  | Recessive | G/G-G/A  A/A | 1  0.00 (0.00-NA) | 0.110 |
|  |  | Over-dominant | G/G-A/A  G/A | 1  0.66 (0.18-2.43) | 0.530 |
|  |  | Log-additive | G/G  A/A | 1  0.44 (0.14-1.37) | 0.140 |
| PTPRO | rs7976329 | Codominant | T/T  C/T  C/C | 1  1.56 (0.48-5.08)  1.56 (0.18-13.11) | 0.750 |
|  |  | Dominant | T/T  C/T-C/C | 1  1.56 (0.50-4.85) | 0.440 |
|  |  | Recessive | T/T-C/T  C/C | 1  1.25 (0.16-9.67) | 0.830 |
|  |  | Over-dominant | T/T-C/C  C/T | 1  1.45 (0.47-4.52) | 0.520 |
|  |  | Log-additive | T/T  C/C | 1  1.37 (0.56-3.36) | 0.490 |
| PTPRO | rs2300290 | Codominant | G/G  A/G  A/A | 1  0.67 (0.20-2.22)  NA (0.00-NA) | 0.150 |
|  |  | Dominant | G/G  A/G-A/A | 1  0.87 (0.28-2.70) | 0.800 |
|  |  | Recessive | G/G-A/G  A/A | 1  NA (0.00-NA) | 0.069 |
|  |  | Over-dominant | G/G-A/A  A/G | 1  0.58 (0.18-1.89) | 0.370 |
|  |  | Log-additive | G/G  A/A | 1  1.18 (0.44-3.15) | 0.740 |
| PTPRO | rs3748299 | Codominant | G/G  G/A  A/A | 1  0.83 (0.24-2.90)  1.11 (0.21-5.80) | 0.930 |
|  |  | Dominant | G/G  G/A-A/A | 1  0.90 (0.28-2.88) | 0.860 |
|  |  | Recessive | G/G-G/A  A/A | 1  1.22 (0.27-5.59) | 0.800 |
|  |  | Over-dominant | G/G-A/A  G/A | 1  0.81 (0.26-2.54) | 0.720 |
|  |  | Log-additive | G/G  A/A | 1  1.01 (0.45-2.23) | 0.990 |
| PTPRO | rs1050646 | Codominant | T/T  C/T  C/C | 1  1.27 (0.36-4.41)  0.00 (0.00-NA) | 0.510 |
|  |  | Dominant | T/T  C/T-C/C | 1  1.11 (0.33-3.75) | 0.870 |
|  |  | Recessive | T/T-C/T  C/C | 1  0.00 (0.00-NA) | 0.270 |
|  |  | Over-dominant | T/T-C/C  C/T | 1  1.33 (0.38-4.62) | 0.650 |
|  |  | Log-additive | T/T  C/C | 1  0.94 (0.31-2.85) | 0.920 |
| PTPRO | rs7956634 | Codominant | T/T  C/T  C/C | 1  NA (0.00-NA)  NA (0.00-NA) | 0.150 |
|  |  | Dominant | T/T  C/T-C/C | 1  NA (0.00-NA) | 0.060 |
|  |  | Recessive | T/T-C/T  C/C | 1  NA (0.00-NA) | 0.270 |
|  |  | Over-dominant | T/T-C/C  C/T | 1  NA (0.00-NA) | 0.120 |
|  |  | Log-additive | T/T  C/C | 1  NA (0.00-NA) | 0.060 |
| PTPRO | rs6488782 | Codominant | C/C  C/A  A/A | 1  0.63 (0.18-2.18)  0.87 (0.05-16.75) | 0.760 |
|  |  | Dominant | C/C  C/A-A/A | 1  0.64 (0.19-2.20) | 0.480 |
|  |  | Recessive | C/C-C/A  A/A | 1  1.19 (0.07-20.21) | 0.900 |
|  |  | Over-dominant | C/C-A/A  C/A | 1  0.64 (0.20-2.11) | 0.460 |
|  |  | Log-additive | C/C  A/A | 1  0.73 (0.25-2.15) | 0.570 |
